# Measuring Co-coverage in Global Health and Nutrition Interventions: A Scoping Review of Analytical Approaches

**DOI:** 10.1016/j.advnut.2026.100742

**Published:** 2026-09-30

**Authors:** Swetha Manohar, Samantha Grounds, Sunny S Kim, Niharika Pandya, Taylor Morrison, Phuong Hong Nguyen

**Affiliations:** 1 Nutrition, Diet and Health Unit, International Food Policy Research Institute, Washington, DC, United States; 2 Department of International Health, Johns Hopkins Bloomberg School of Public Health, Baltimore, MD, United States

**Keywords:** co-coverage, coverage, nutrition and health interventions, multisectoral nutrition, coverage measurement, maternal and child nutrition

## Abstract

Multisectoral nutrition (MSN) approaches coordinate interventions across sectors, such as health, agriculture, education, and social protection, to improve nutrition outcomes and have gained prominence in low- and middle-income countries (LMICs). Measuring co-coverage—the clustering of multiple interventions at the individual and household level—remains uncommon yet offers a viable method for tracking MSN intervention coverage. This scoping review examined existing analytic approaches and applications for assessing the co-coverage of health and nutrition interventions. We searched Web of Science Core Collection, PubMed, and EBSCOhost for studies that used, described, or developed co-coverage methods to measure nutrition- and health-related intervention coverage. Titles and abstracts were screened by 2 independent reviewers (SG and NP) using Covidence, followed by full-text screening based on predefined criteria. Screening 4478 abstracts yielded 73 eligible studies applying 8 distinct methods to quantify co-coverage across health and nutrition interventions. Most studies were observational (90%) and conducted in LMICs, particularly sub-Saharan Africa and South Asia, with publications increasing over the past decade. Data were primarily drawn from surveys (93%), particularly Demographic and Health Surveys (41 studies). Commonly included individual level interventions in co-coverage indices were antenatal care (ANC, 66 studies) or ANC components, child immunizations (48 studies), and skilled birth attendance (42 studies). Evidence on measuring a range of multisectoral interventions at the household level was limited. Intervention selection was often guided by established frameworks or guidelines. Coverage indices were calculated using simple counts, weighted averages, and scoring systems. Although 18 studies validated their indices through methods such as Cohen’s κ and sensitivity analyses, most relied on previously used approaches without specific validation. These findings underscore the considerable variability in co-coverage methodologies and the need for greater harmonization and validation to enable consistent monitoring of multisectoral nutrition progress.

**Statement of significance**

Existing literature on co-coverage analysis predominantly focuses on child-survival interventions and the 1000-day period for women and children at the individual level. Less is known about how these methods can be leveraged for use in assessing the co-coverage of multisectoral nutrition integration at the household level or across other life stages, including late childhood and adolescence. Clarifying and consolidating available co-coverage methodologies and their practical application can guide the adaptation of existing approaches and/or to develop new analytic methods to track integrated nutrition programs for different target populations.

## Introduction

Malnutrition persists as a global challenge and a multifaceted issue. Globally, an estimated 150 million children under 5 were stunted, 43 million wasted, and 35.5 million overweight [1], while unhealthy diets account for 20% of all adult deaths globally [2]. In response, multisectoral nutrition strategies that coordinate efforts across the health, agriculture, education, water, sanitation, and social protection sectors have gained political traction and funding to accelerate progress toward the 2030 Sustainable Development Goals [3,4]. Implementing these strategies requires coordinated efforts across sectors and life stages to improve diets and nutrition outcomes among vulnerable populations, particularly in LMICs [5,6].

To track the progress of scale-up, reach, and effectiveness of interventions outlined in these strategies, it is essential to measure coverage—defined as “the proportion of the target population in need of interventions who actually receives them” [7]. Measuring coverage for multisectoral interventions is challenging because of diverse delivery platforms (e.g., community, facility, household, and social protection), varied target populations (e.g., infants, pregnant women, and adolescents), and the absence of standardized valid coverage measures for many nutrition-sensitive interventions such as school feeding, food fortification, and nutrition-sensitive agriculture interventions [8].

The traditional approach to measure coverage analyzes each intervention separately and then stratifies by factors such as gender or socioeconomic position [9,10]. However, single-indicator analyses provide only a partial view, failing to capture how multiple interventions reach the same populations. These limitations underscore the need for approaches that assess multiple interventions across sectors simultaneously. Co-coverage is one such method for measuring the receipt of multiple interventions at the individual or household level, whereas composite coverage is typically calculated at higher administrative or group levels, such as districts, provinces, or population subgroups [11,12]. In this review, we focus on co-coverage because it captures the clustering of interventions within individuals and households, provides insights into integration across sectors, identifies inequities, and informs policy and programmatic decision making through resource allocation, targeting, and service delivery improvements [13]. It can be expressed in terms of both the proportion of individuals receiving multiple interventions (magnitude) and the mean number of interventions received (comprehensiveness), providing complementary perspectives on program reach and integration.

Existing literature [12,[14], [15], [16]] describes several approaches for estimating co-coverage. These approaches include measuring co-coverage by population or life stage (e.g., mother–child dyads), by specific program type or problem (e.g., vitamin A or anemia control programs). Another possible approach to measuring co-coverage could be by multisectoral integration (e.g., nutrition-sensitive social protection, which includes cash transfers along with health and/or nutrition interventions for pregnant and lactating women). However, to date, co-coverage analyses have predominantly focused on health sector interventions during the first 1000 days of life [11,13,17]—a reflection of the method’s original use in tracking maternal mortality prevention and child-survival interventions in global health—leaving significant gaps in understanding of how these methods can be applied to other life stages or for broader multisectoral nutrition programs. Expanding co-coverage analyses beyond health sector interventions to include nutrition interventions implemented by other sectors, such as water and sanitation or school feeding interventions, could strengthen assessment of multisectoral program delivery and inform the adaptation of existing methods and/or inform the development of new co-coverage analytic methods, as needed.

To fill this gap, we conducted a systematic scoping review to examine analytical methodologies for measuring the co-coverage of multiple nutrition- and health-related interventions. We assessed methodological approaches, limitations, and practical applications across contexts. This review addresses 3 research questions: *1*) what approaches have been used for selection and prioritization of interventions to include in co-coverage analysis; *2*) what existing analytical methodologies have been used for assessing co-coverage and their considerations and trade-offs; and *3*) how have the methodologies been applied and validated across different contexts and settings? The findings from this review aim to inform future efforts to measure and monitor co-coverage of multisectoral nutrition, health, and related interventions, guide the development of transparent and validated indices, and promote alignment with national nutrition strategies to improve tracking, decision making, and accountability.

## Methods

### Study design

The systematic scoping review was guided by a protocol using PRISMA extension for Scoping Reviews (ScR) guidelines [[18], [19], [20]]. The scoping review method was well-suited to examining a broad range of studies, definitions, and conceptual boundaries of co-coverage and its application across various contexts.

### Search strategy

We searched PubMed, Web of Science Core Collection, and EBSCOhost for studies published in English between 1990 and 2024, including book chapters. The search strategy was developed using an iterative process around 3 key themes: *1*) the topic of interest, that is, “coverage” and/or “co-coverage”; *2*) the fields of interest, i.e., health and/or nutrition; and *3*) the metrics of measurement, such as index, weighting, score, etc. After performing exploratory searches and undergoing librarian and topical expert consultation, a comprehensive search strategy was finalized. Search terms were combined using Boolean operators, for example (“co-coverage” OR “coverage” OR “multiple interventions”) AND (“nutrition” or “health”) AND (“analytic methods” OR “methods” OR “analysis” OR “metrics” OR “weighting”). Variations of the search string were adapted for the different databases. Proximity operators were used for themes 1 and theme 3 to improve search precision. An example of a final search string can be found in Supplementary Methods 1. The search (conducted in May 2024) and subsequent data extraction and coding were informed by operational definitions (Supplementary Methods 2) for key terms based on seminal literature, described in the next section.

### Inclusion and exclusion criteria

Studies were eligible for inclusion if they met the following criteria (Supplementary Methods 3): *1*) covered the general population; *2*) measured intervention coverage at the population-, household-, dyad-, or individual level; *3*) included health and/or multisectoral nutrition intervention coverage [21] (full list in Supplementary Methods 4); *4*) evaluated co-coverage as an exposure or outcome and composite coverage only if calculated from a single data source; *5*) used quantitative or mixed-methodologies; and *6*) study designs including experimental or observational studies using either primary or secondary data from a single data source. An exception for inclusion was made if data came from different sources but were able to be linked at the individual level (e.g., health card data linked with individual-level data).

Studies were excluded if they focused on animals, single interventions, qualitative methods only; were meta-analyses, reviews, commentaries/op-eds, or conference abstracts; computed coverage using multiple sources or aggregate measures; or were published before 1990 or without full-text available in English.

The terms co-coverage and composite coverage are sometimes used interchangeably. The operational definition of co-coverage utilized to guide this review was based on seminal literature on the topic and defined as a metric that measures the coverage of multiple recommended nutrition-related interventions received by a target population at the individual or household level [11,16]. In this review, co-coverage was distinguished from composite coverage. Composite coverage was defined as the population-level measurement of coverage across multiple nutrition and health interventions, typically aggregated at the group level using multiple data sources [11]. Studies using composite coverage metrics meeting this definition were excluded. However, studies reporting composite coverage indices like the composite coverage index (CCI) were included, as noted above, if estimates were derived from: *1*) household-level coverage data collected via household surveys (not summaries/reports) and *2*) individual-level coverage data, as typically, the CCI aggregates individual-level receipt of interventions to construct a composite indicator. Henceforth, when we refer to co-coverage indices/measures included in this review, this includes the composite indices that met the criteria detailed above. The full inclusion and exclusion criteria are provided in Supplementary Methods 3.

### Data extraction and analysis

Search results were imported into Covidence, a web-based platform to support the conduct of reviews, for screening and data management. Duplicate records were removed. Title and abstract and full-text screening were independently conducted by 2 reviewers (NP and SG). Conflicts were resolved by a third independent reviewer (SM) with input from the study team, as needed. A data extraction template was prepared and pretested on 5% of included studies (Supplementary Methods 5). The extracted data included study characteristics (title, publication year, author, first author affiliation, journal, and article type); study region, country, and broad socioeconomic context; study design/type (observational or experimental); study method (quantitative or mixed methods); other characteristics: urbanicity, humanitarian context; relevant study population [infants, children aged <2 y and 5 y, women of reproductive age (15–49 y), pregnant and lactating women] and sample size; study aims and outcomes; and data sources for the study’s co-coverage measurement(s) (type, source, and whether multiple rounds of data were used). Data types were split into survey data or records or administrative data (such as health center records and antenal care cards), direct observation, and other (multiple data sources). Specific to the method under study, data were extracted on the analytic methods, co-coverage index/metric/indicator used (name, selection/prioritization of interventions used to construct the index, unit of analysis, method of calculation, interventions considered in its construction, number of interventions used, validation methods, applications of the indicator, and the limitations of the indicator). Data on the selection/prioritization of interventions used to estimate co-coverage were extracted based on reasons either explicitly stated by the authors of the included studies or inferred by the reviewers through examination of the text. Data extraction was carried out by 1 reviewer, and a subset was reviewed for accuracy and completeness by a second. A third reviewer conducted targeted sensitivity checks to ensure consistency. Methodological quality appraisal of the studies was not performed for this review [19]. Details of data extraction procedures, codes, and their definitions, and how codes link to each aim can be found in Supplementary Methods 5. Study characteristics were summarized in tables, and visualizations were created to show the interventions included in the co-coverage indices and the validation methods used by different indices. The key trends in methods used to develop co-coverage indices are consolidated in tabular format and narratively described.

## Results

The study selection process for this review is shown in the PRISMA flowchart (Figure 1 [[22], [23], [24], [25], [26], [27], [28], [29], [30], [31], [32], [33], [34], [35], [36], [37], [38], [39], [40], [41], [42], [43], [44], [45], [46], [47], [48], [49], [50], [51], [52], [53], [54], [55], [56], [57], [58], [59]]). A total of 5188 studies were identified across the 3 databases, of which 4478 titles and abstracts were screened after duplicates were removed. Of these, 112 were retained for full-text screening. Studies excluded at this stage used composite indicators or aggregated means to compute coverage indicators or evaluated the quality of coverage of a single intervention. Composite indicators were excluded because they are calculated at higher administrative or group levels (e.g., districts, provinces, or population subgroups) rather than at the individual or household level, making them conceptually distinct from co-coverage indicators. One study was excluded because the full-text article was unavailable in English. Ultimately, 73 studies met the inclusion criteria for the study.

**FIGURE 1:**
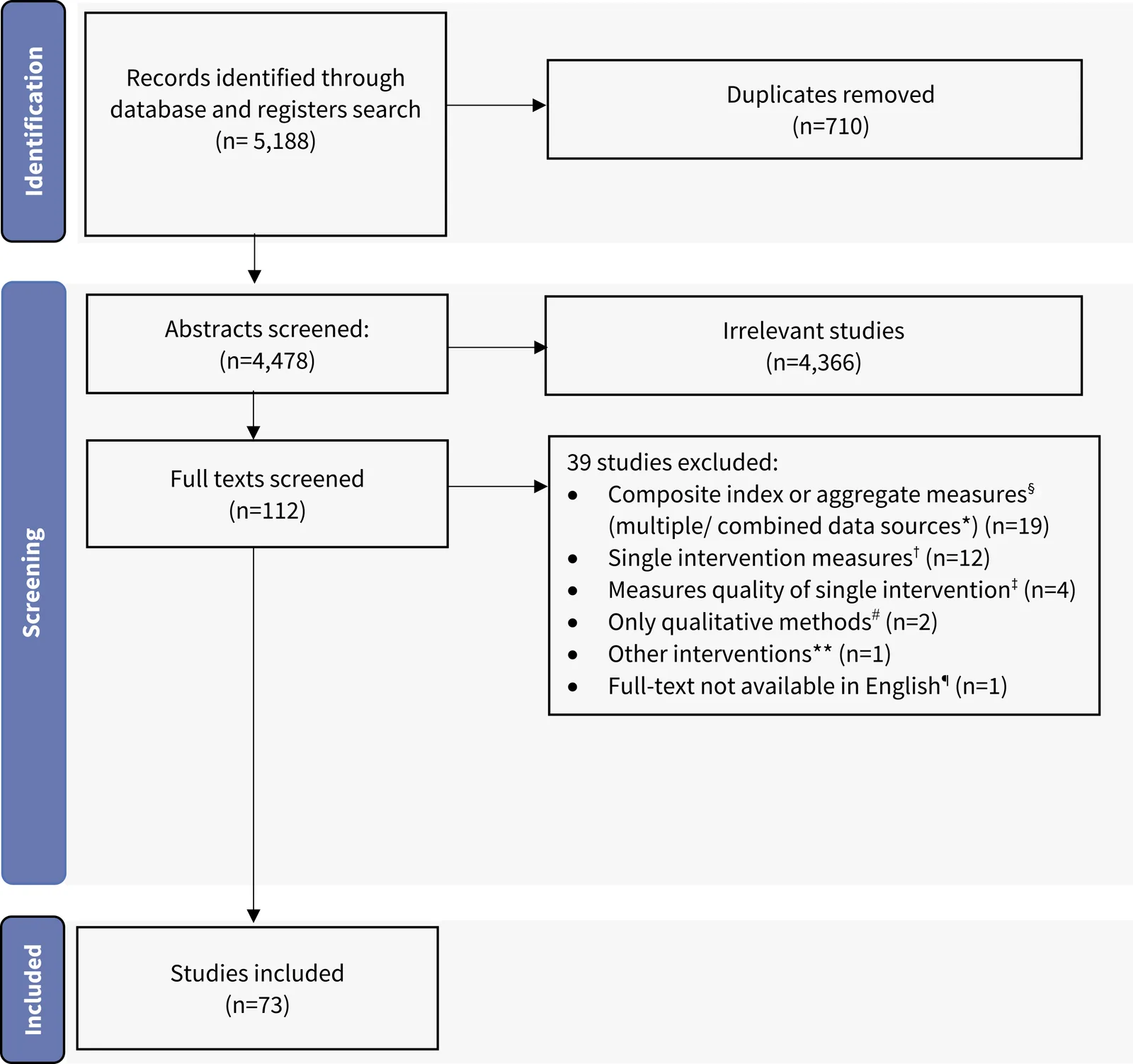
PRISMA flow diagram. Data from facility-level data and household data combined. ^§^Composite index or aggregate measures excluded: [[22], [23], [24], [25], [26], [27], [28], [29], [30], [31], [32], [33], [34], [35], [36], [37], [38], [39]]. ^†^Single intervention measures: [[40], [41], [42], [43], [44], [45], [46], [47], [48], [49], [50], [51]]. ^‡^Measures quality of single interventions: [[53], [54], [55], [108]]. ^#^Only qualitative methods: [56,57]. ∗∗Other interventions: [58] ^¶^Full test not available in English: [59].

### General study characteristics (study setting, context, and design)

Summary statistics presented here are not additive to the total number of studies included (*N =* 73), as many studies included multiple domains, measures, populations, and settings. Most studies included data from sub-Saharan Africa (*n =* 40), followed by South Asia (*n =* 36). Far fewer studies focused on Europe and Central Asia (*n =* 7) and none from North America (Figure 2A and B). Among the 73 studies, 67% (*n =* 49) focused on 1 country, whereas 33% (*n =* 24) measured intervention coverage in multiple countries, with ≤83 countries being considered in 1 study [60]. Using World Bank definitions [61], LMICs were represented most frequently (*n =* 67), followed by upper-middle-income countries (*n =* 24) and high-income countries (HIC) (*n =* 8). All the HIC were from Latin America and the Caribbean region (Figure 2A).

**FIGURE 2:**
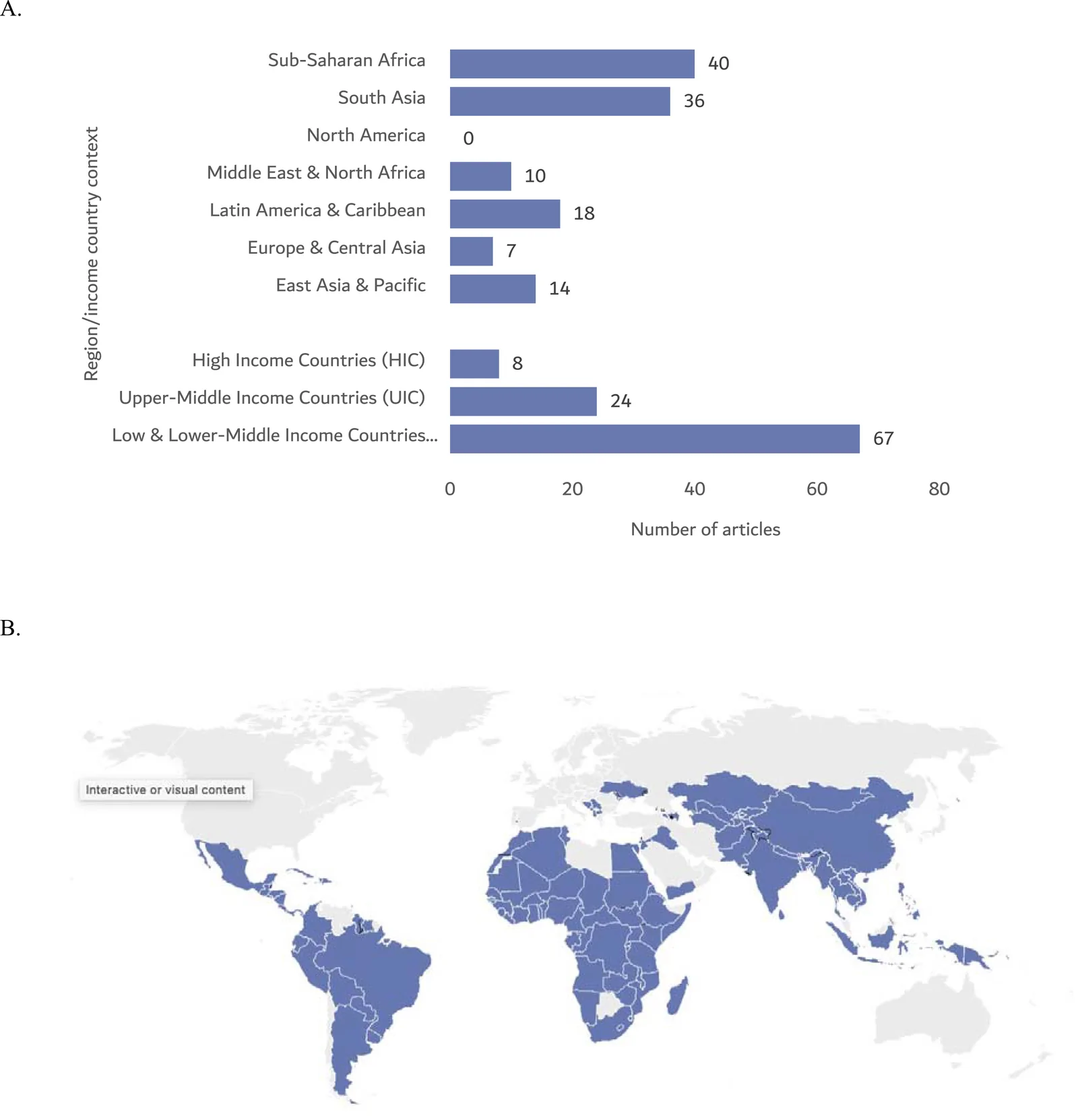
Distribution of geographic and country-income classification of included studies. (A) Bar graph of studies by World Bank country-income classification and regions. (B) Choropleth map of the countries represented across included studies. Some studies covered >1 country.

All included studies were research studies, except for one, which was a study protocol [62]. There was an increasing trend over time in the number of studies published on co-coverage, with only 2 studies published between 2005 and 2009 compared with 41 studies published in the last 5 y, from 2020 to 2024. The majority (*n =* 66) of the studies were observational studies, with only 7 studies utilizing experimental or quasi-experimental approaches. Key study characteristics are outlined in Supplementary Table 1, and the list of included studies in Supplementary Table 2.

For the indices of interest, most studies (*n =* 68) used primary data—specifically, survey data. Primarily, the data source to construct the co-coverage indices were population-based surveys, especially Demographic and Health Surveys (DHS) (*n =* 41). Multiple Indicator Cluster Surveys (MICS) (*n =* 16), and other national surveys (*n =* 16) . Some studies that focused on multicountry studies used both DHS and MICS data from multiple countries, using the most recent survey available in each country (Supplementary Table 1). There were only 5 studies that combined nonsurvey and survey data, specifically data from health records, antenatal care (ANC) cards, census, or direct observation [[62], [63], [64], [65], [66]]; these data were combined with individual level household data to compute the co-coverage indices. Although we excluded papers that combined multiple data sources, 3 studies (a subset of the 5 that used nonsurvey data) were included that used multiple data sources that pertained to the same individual. These included primary data collected through a survey that included both face-to-face interviews at the individual level and direct observation at the household level (the only study to use mixed methods) [62], and survey data collected from women receiving ANC with observed practices during ANC visits and data from ANC cards [62,64]. Another study used multiple data sources (nonadministrative data), combining survey data from: *1*) a surveillance study; *2*) a survey on financial coverage; and *3*) a survey on ANC and PNC that were able to be linked based on the availability of unique individual identifiers [63].

Overall, 50 studies were at the national level, with no specific focus on a particular residence context (urban, periurban, and rural). Of the remaining studies, 17 reported a rural context, 1 reported a periurban context, and 8 reported an urban context. Only 3 studies focused on a humanitarian setting [60,67,68].

Target populations representing the denominators of co-coverage index calculations are shown in Supplementary Figure 1. Some coverage indices only comprise one population of interest (e.g., pregnant and lactating women), whereas others included multiple populations (e.g., children aged <2 y, children aged <5 y, pregnant and lactating women, and women aged 15–49 y). The majority of the articles focused on indicators relevant to pregnant and lactating women (*n =* 70) and women of reproductive age (*n =* 61). A few more studies captured pregnant and lactating women because they specifically outlined recruiting women who were pregnant or recently delivered as their target groups without age limits. As a result, 9 articles were not coded as including women aged 15 to 49 y in their target population, even though these target populations likely fell within that age range. Indicators for children aged <5 y (*n =* 36) and children aged <2 y only (*n =* 39) were also often included. Overall, there was a high overlap between studies that included women and children as the target population with related mother–child dyad intervention (co-)coverage measured. A few studies (*n =* 4) included other populations of interest such as patients who used inpatient services or people with a certain chronic disease [69] or measured coverage at the household level [70].

### Approaches for selection and prioritization of interventions for co-coverage measurement

Several interventions were selected for inclusion in different combinations to estimate co-coverage. The indices were estimated at the individual (*n =* 49; of which 30 studies computed the CCI), mother–child dyad (*n =* 28, of which 7 computed the CCI), or household level (*n =* 1). Studies that estimated the CCI using individual-level data (also coded as “Other” for the code domain “unit of analysis”) were included to offer insights on interventions to include in co-coverage metrics.

The most frequently included coverage intervention indicators used to measure co-coverage were ANC by a skilled provider (*n =* 66) and immunizations for children (*n =* 48); primarily Bacille Calmette-Guerin (BCG), diphtheria tetanus toxoid and pertussis (DTP), and measles vaccination. Figure 3 illustrates a range of interventions used in co-coverage measures, grouped by life stage (for individual-level interventions) and by household-level interventions. Indicator definitions varied by study, often aligning with DHS definitions given their widespread use, although in some cases, definitions were based on available data and how it was collected. For example, the criteria for ANC coverage being met were different across studies. In some analyses, ANC coverage was met if any ANC with a skilled provider was provided [70]; in others, if at least ANC visits with a skilled provider were complete [71]; and, in others, frequency of ANC was considered for coverage to be counted [72]. Other common interventions included skilled birth attendance at delivery (*n =* 42), contraception/family planning as part of PNC (*n =* 30), tetanus toxoid immunization during pregnancy (*n =* 27), and blood and urine (*n =* 22 each) checks and blood pressure assessment (*n =* 20) during ANC. Although less common, certain indicators included in the co-coverage index measured practices rather than interventions, such as early initiation of breastfeeding [73,74].

**FIGURE 3:**
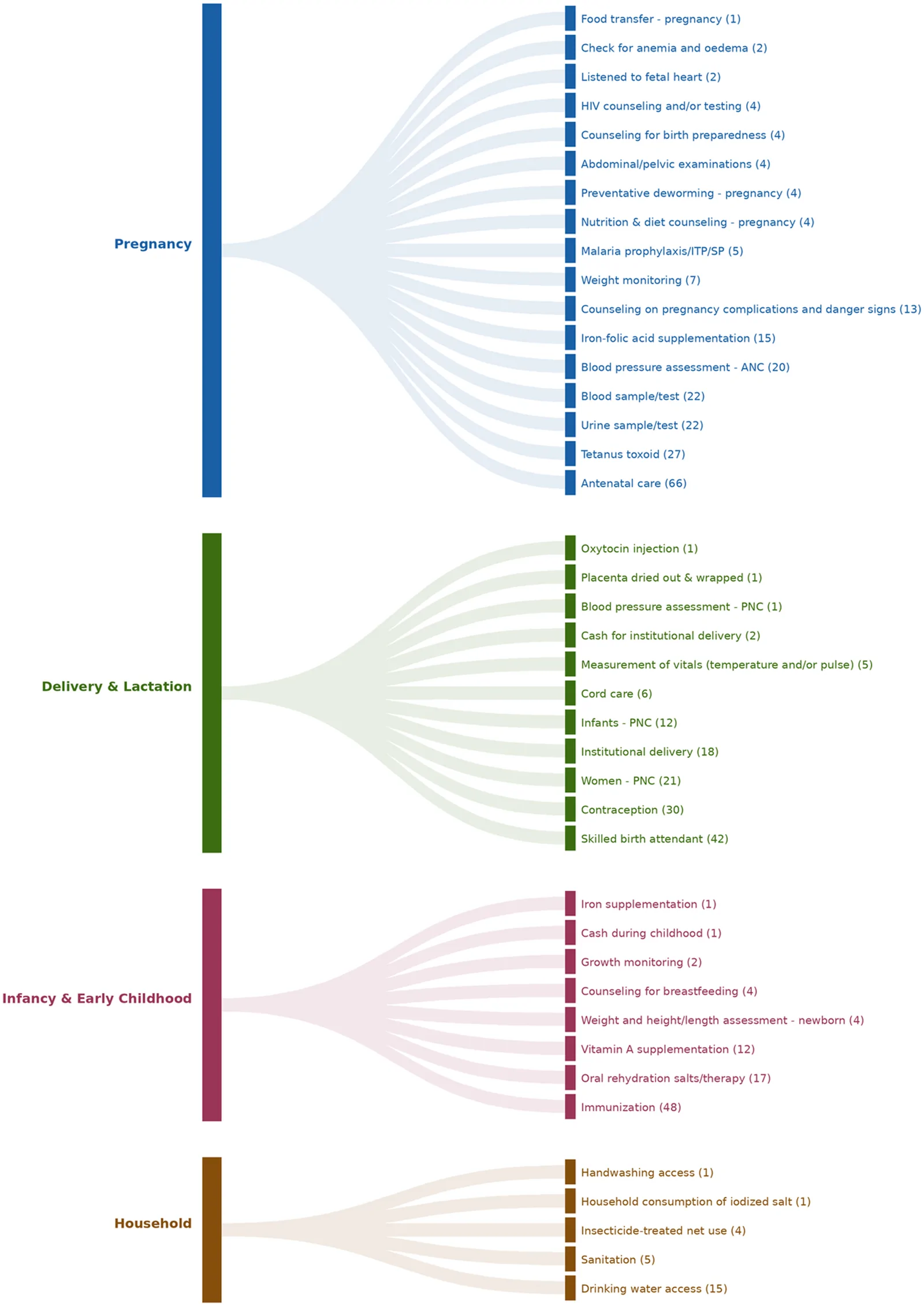
Sankey diagram illustrating the number of studies linking coverage indicators (or interventions) to the key populations for which they are measured.

Among interventions directed at the household, access to safe drinking water was a commonly included intervention (*n =* 15); other household interventions, such as sanitation (e.g., access to an improved toilet, household cleanliness) (*n =* 5), use of insecticide-treated nets (*n =* 4), and access to handwashing facilities (*n =* 1), were included less frequently than individual-level maternal and child interventions focused primarily on the first 1000 days of life (Figure 4). All papers except 1 [68] offered a stated rationale for including interventions in their estimation of co-coverage, with many stating multiple reasons for intervention selection. Most papers (*n =* 67) prioritized essential preventive or curative interventions related to specific life stages. Over half of the studies (*n =* 41) stated that they referred to existing literature measuring coverage to prioritize which interventions to select. One-third of the studies (*n =* 27) employed methods developed by the Countdown Initiative—including methods used in the Countdown to 2008 [75], 2015 [76], and 2030 reports [13]— which encompass the original [16] and updated CCI [77], and the co-coverage measure of preventive interventions proposed by Victora et al. [14].

**FIGURE 4:**
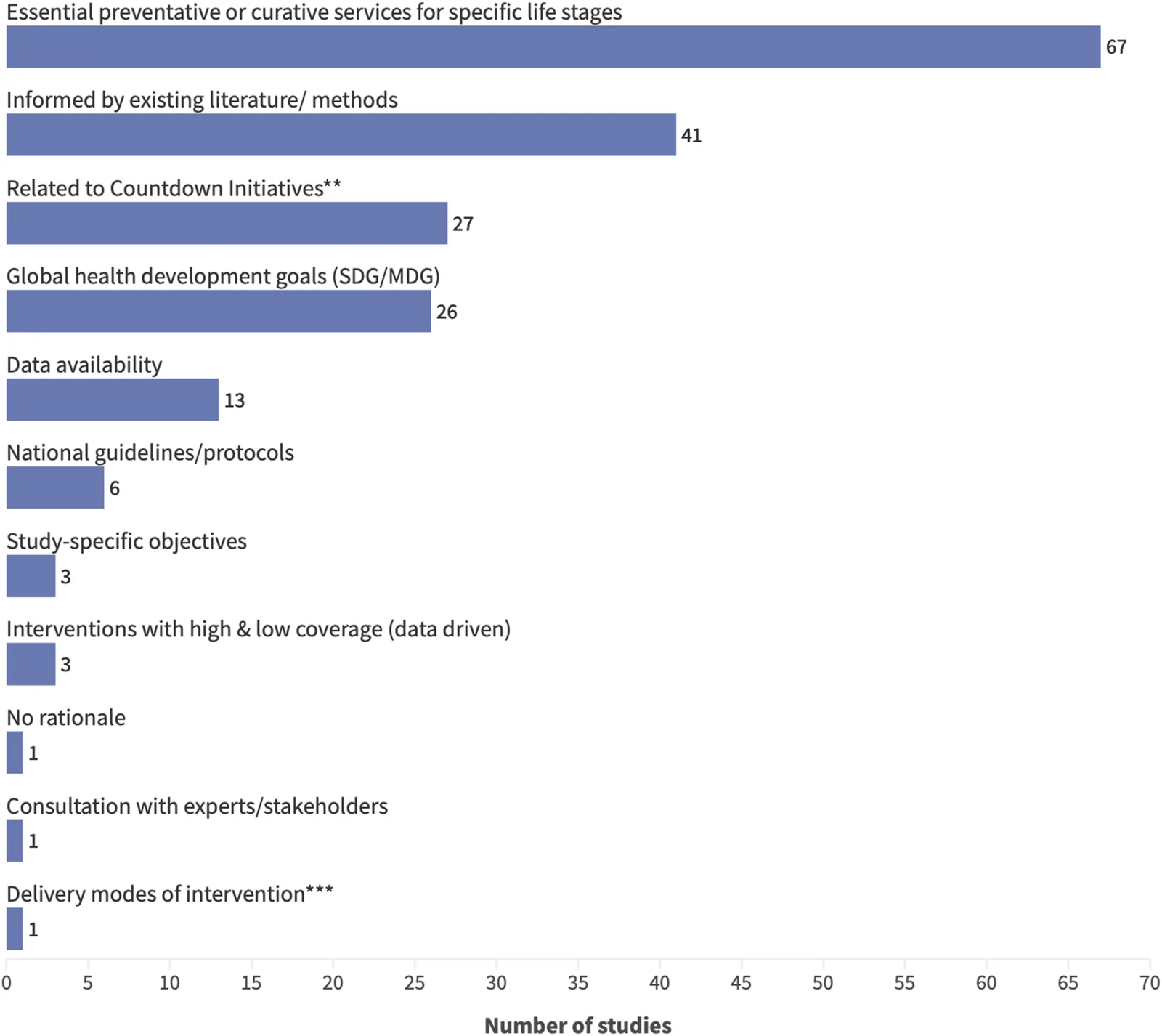
Approaches used to select and prioritize interventions. MDG, Millennium Development Goal; SDG, Sustainable Development Goal. Studies may have reported multiple approaches to selecting and prioritizing interventions. Studies that reported using Countdown to 2030n initiative methods were coded as being related to global health development goals. Delivery modes included service providers, health system, community-based, campaigns.

The literature included in the review draws on existing frameworks for indicator selection including the World Bank and World HealthOrganization’s (WHO) monitoring framework toward universal health coverage (UHC) [63,69,[78], [79], [80]], the Lancet Global Health Commission on High Quality Health Systems (HQSS) framework [[81], [82], [83]], and WHO’s World Health Report 2010 focused on health systems financing for UHC [69,84,85], and conceptual frameworks from other sources [86]. Many of the frameworks above leveraged elements of the continuum of care, and some studies applied the concept directly to inform intervention selection. The literature underpinning this approach included the WHO continuum of care framework by Kerber et al. [71,87], WHO recommendations for ANC [83,88] where in some papers they focused only on mothers [72] and WHO’s Safe childbirth checklist and Integrated Management of Childhood Illnesses (IMCI) guidelines [82,89].

Some studies, such as Kim et al. [83], drew on country policies as well as multiple guidance documents to inform intervention selection. This included the WHO’s Standards for Improving the Quality of Care for Children and Young Adolescents in Health Facilities, WHO’s recommendations for ANC, the HQSS framework, and the study country’s national sexual and reproductive health policy. The final set of interventions included, even if informed by the literature, were sometimes limited or modified by data availability (*n =* 13). In a few cases, the data availability appeared to be one of the key criteria for the choice of indicators to include in the estimation of co-coverage (*n =* 13) [69,78,90,52] (collapsed as “data availability” in Figure 4).

### Existing analytical methodologies used for co-coverage assessments (considerations and trade-offs)

There were 8 distinct aggregation methods reported to estimate co-coverage; the most frequently reported was a simple count (or summation) of interventions (*n =* 36) received at the individual or dyad level (Table 1 [10,11,12,14,16,52,60,[63], [64], [65], [66], [67], [68], [69],[71], [72], [73], [74],^77^,^78^,^80^,^82^,^83^,^85^,^86^,[90], [91], [92], [93], [94], [95], [96], [97], [98], [99], [100], [101], [102], [103], [104], [105], [106], [107], [108], [109], [110], [111], [112], [113], [114], [115], [116], [117], [118], [119], [120], [121], [122], [123], [124], [125], [126], [127], [1]]). The transformed index generated using summation was then used to: *1*) report co-coverage as a continuous outcome at the population level, *2*) create a categorical outcome with thresholds for “high” or “low” co-coverage, or *3*) create a binary (“all or nothing”) outcome where a “1” was applied if all interventions were received to indicate co-coverage achieved and 0 if not all interventions were received. A related, but less common, method was a scoring method (*n =* 2) [66,108]. For this approach, the index was still calculated by adding up the different interventions received, but different interventions contributed unequal point values to the total score (e.g., having ≥1 ANC visit with a skilled provider was scored as 2 points, while having blood pressure measured during ANC was worth 1 point) [108]. Many authors also calculated a weighted linear mean (*n =* 33), often using the CCI (*n =* 27) [11,52,60,67,71,72,77,85,89,92,[109], [110], [111], [112], [113], [114], [115], [116], [117], [118], [119], [120],[123], [124], [125], [126], [127],^129^] or coverage gap index (CGI) methodology (*n =* 4) [121,122,130,131] or modifying the approach to include indicators of interest. In these cases, the weighted average was applied to coverage for specific populations compared with that at the individual level—essentially an aggregated approach. Other reported calculation methods included computing a linear mean (*n =* 5) [73,82,85,92,91], geometric mean (*n =* 2) [69,80], using principal component analysis (*n =* 2) [69,128], performing regression weighting (*n =* 1) [69], and using a percentage difference method (*n =* 1) [68] (Table 1 for a summary of methods used for indices and Supplementary Table 3 for a detailed breakdown).

**TABLE 1:** Methods used to construct co-coverage indices^1^

Analytic approach	Index estimation (formula) (illustrative example)	Studies	Index creation (illustrative example)	Unit of analysis^2^	Life stage	Sectors considered
Cumulative count of interventions received (*n =* 36)	Sum of multiple interventions measured at the individual level For example, > 1 ANC visit + tetanus vaccination during pregnancy + skilled birth attendant during birth + BCG vaccination + measles vaccination + childhood VitA supplementation + access to improved drinking water in household [11]	[12,14,65,71,74,16,82,83,86,90,91,92,93,94,10,63,[95], [96], [97], [98], [99], [100], [101], [102], [103], [104], [105], [106], [107]]	• Score range based on data availability, for example, 0–8 • Categories created by applying cutoffs, e.g., ≥6 = high • Classification of extent of coverage: • High coverage: ≥6 interventions • Medium coverage: 3–5 interventions • Low coverage: 0–2 interventions [102]	• Mother–child dyad • Individual	• Pregnancy • Delivery • Childhood • Household	• Health • Nutrition • WASH
Scoring (*n =* 2)	Score generated based on a set of interventions that are recommended at a certain life stage. Points are assigned based on intervention receipt; total score calculated. For example, ANC score: antenatal visits = 5 points (30 points); for 6 visits; 1 pt for each laboratory test, immunization, prescription, hepatitis complete blood count/total maximum: 42 points [66].	[66,108]	• Dichotomous indicator generated: • “High” ANC score if ≥42 points • “Low” ANC score if <42 points	• Individual	• Pregnancy	• Health • Nutrition
Linear mean (simple average) (*n =* 5)	Average of multiple recommended interventions based on life stage or other norms and policy For example: MCH Service Coverage = average of ANC 4+, facility delivery, and care seeking for child diarrhea, pneumonia, and fever [73]	[73,82,85,91,92]	• Continuous score	• Individual • Mother–child dyad	• Pregnancy • Delivery • Childhood	• Health • Nutrition
Weighted linear mean (*n =* 33)	Interventions weighted by importance or frequency. For example: Modified Composite Coverage Index: 1/3((2∗DTP3 + MSL + BCG) + (VitA + TT + ANC/3) + (SBA + AIW/2) [92]	[11,60,67,[71], [72], [73],^16^,^77^,^78^,^85^,^90^,^52^,[109], [110], [111],[112], [113], [114], [115], [116],^117^,^118^,^119^,^120^,^121^,^122^,^92^,[123], [124], [125], [126], [127]]	• Continuous score	• Individual • Mother–child dyad	• Pregnancy • Delivery • Childhood	• Health • Nutrition
Geometric mean (*n =* 2)	Geometric mean of selected interventions (*n*) based on life stage or to achieve universal health coverage For example: UHC Index (service coverage) geometric average of service coverage (breast/cervical cancer screening, >4 ANC, PNC, immunization, EBF, safe drinking water, adequate sanitation, physical access to health facility, nonuse of tobacco, cesarean section rate, hospital delivery, SBA, use of outpatient care, hospital admissions) and financial protection (health insurance coverage, no self-discharge for financial reasons, no catastrophic health expenses) [80].	[69,80]	• Continuous score	• Individual	• Pregnancy • Delivery	• Health • Nutrition • WASH • Social protection
Principal component analysis (*n =* 2)	*PCA, i.e., linear dimensionality reduction technique used to generate co-coverage index from* dichotomous indicators and *sociodemographic factors*. For example: ANC component coverage index, which included the following dichotomous indicators: women’s weight, height, and blood pressure, fundal height, fetal heartbeat, blood test for HIV and syphilis, external and internal pelvic examination, receiving 90 IFA pills, 2 tetanus toxoid injections, information on pregnancy complication signs and what to do if there are signs, child sex, first child, mother’s education, mother’s age, monthly household expenditure, and asset ownership [128].	[69,128]	• Continuous score	• Individual	• Pregnancy • Delivery	• Health • Nutrition
Regression weighting (*n =* 1)	Regression weighting creates a composite score by applying regression-derived coefficients to multiple indicators, weighting each based on its estimated contribution to an overall outcome. For example, *b*_0_ + (full ANC∗*b*_1_) + (institutional delivery∗*b*_2_) + (postnatal visits∗*b*_3_) + (full immunization∗*b*_4_) + (contraceptive prevalence rate∗*b*_5_) + (ORS use rate∗*b*_6_) + (curative care from qualified provider∗*b*_7_) + (met need curative care∗*b*_8_) + (poverty impact (@$1.25 (PPP))∗*b*_9_) + (catastrophic health expenditure∗*b*_10_) where CS = composite score *b*_0_ = constant, *b*_1_, *b*_2_, *b*_10_ = regression coefficients (or weights)	[69]	• Continuous score	• Individual	• Pregnancy • Delivery	• Health
Percentage difference (*n =* 1)	Percentage difference estimated to compute the absolute value of the change in coverage between 2 time points. Resilience index (co-coverage index) constructed using the percentage change in coverage across 14 MNCH indicators between 2 timepoints (2011 and 2015) Indicators included: contraceptive prevalence, 4+ANC, 2+ tetanus toxoid vaccination during last pregnancy, IPT2 malaria prophylaxis, SBA, 1+ postnatal visit, slept under LLIN/ITN night of survey, VitA, DTP3, full vaccination, <5 y slept under LLIN/ITN night of survey, <5 y diarrhea treatment with ORS, U5 ARI treatment with appropriate antibiotics, and <5 y fever treatment with appropriate antimalarial	[68]	• Continuous score • Score between 0 and 1.	• Individual	• Pregnancy • Childhood	• Health

Abbreviations: ANC, antenatal care; ANC4, 4 or more antenatal care visits; ARI, acute respiratory infection; BCG, Bacille Calmette-Guerin vaccine; CS, composite score; DTP3, diphtheria, pertussis, and tetanus vaccine; EBF: Exclusive breast feeding; FP, family planning; HB3, hepatitis B vaccine; IFA, iron folic acid; IPT2, intermittent preventive treatment; ITN, insecticide-treated net; LLIN, long-lasting insecticidal net; MSL, measles vaccination; MCH, maternal child health; OGTT, oral glucose tolerance test; ORS, oral rehydration salts; PCA, principal component analysis; PLO3, Polio vaccination; PNC, postnatal care; PPP, purchasing power parity; RMNCH, reproductive, maternal, newborn, and child health; SBA, skilled birth attendant; TT, tetanus; TDAP, tetanus, diphtheria, and pertussis vaccination; <5 y: under 5 years of age; UHC, universal health coverage; VitA, vitamin A,WASH, water, sanitation, and hygiene.

1 Expanded table in Supplementary Table 3.

2 Individual: mother–child dyad.

For the unit of analysis, most studies calculated co-coverage indices with analysis at the individual (*n =* 49) or mother–child dyad level (*n =* 28) (Table 1 and Supplementary Table 3). Some of the indices computed at the individual or dyad level did include household-associated variables, specifically indicators related to access to safe water, improved sanitation, and handwashing facilities, but they were coded as being assessed at the individual or dyad level, respectively. Only 1 study explicitly measured coverage at the household level [70], and these indicators included interventions focused on safe water and sanitation, reflecting sectors beyond health and nutrition. A total of 30 studies were additionally coded as “other”; these studies mostly computed the CCI (and sometimes the CGI and UHC) but used data measured at the individual level. These overlapped with 24 and 7 studies that assessed co-coverage at the individual and mother–child dyad levels, respectively.

Limitations related to the co-coverage indices’ construction, use, and interpretation were examined as reported by authors of the studies included in the review (Supplementary Table 2). Many authors (*n =* 21) reported inadequate information and measures on the quality, timing, and content of interventions, limiting the ability to comprehensively measure co-coverage and include a full range of recommended interventions. Some author groups also reported missing data (*n =* 5) as being a limitation to compute the index more comprehensively as well as the inability to include some interventions of interest due to measurement error (*n =* 1) [78]. Because of a heavy reliance on survey data to construct co-coverage indices, key methodological weaknesses of cross-sectional population-based surveys were also raised as limitations. For example, surveys had long recall periods of 2, 3, or 5 y, and 48% of author groups (*n =* 35) reported the potential for recall bias for those indicators. This limitation also poses a threat to the validity of those co-coverage measures. Also related to self-reported data were concerns about social desirability bias (*n =* 7) and respondents’ comprehension of survey questions and care received (*n =* 3). Less frequently mentioned—but still relevant—were issues such as inaccuracies in ANC cards (*n =* 1) and inter-interviewer variability (*n =* 1). Other limitations identified included restrictive sample sizes to measure co-coverage when the target population is pregnant women because the index includes indicators measured only among women with births specifically in the last 2 y (*n =* 2). Limitations in the use and interpretation of co-coverage indices to measure coverage were also noted. Some studies (*n =* 2) found that presenting co-coverage alone—without showing the individual intervention coverage rates—fails to reveal which interventions are improving or declining, which limits actionable insight from the indices [77,52]. Additionally, 1 study pointed out that the co-coverage index did not detect bottlenecks in care delivery [60]. All limitations are listed in Supplementary Table 4.

### Application and validation of co-coverage measurement across different contexts and settings

Co-coverage indices were most often applied to examine inequities and inequalities in coverage (*n =* 47), followed by monitoring intervention coverage (*n =* 36) (Figure 5). “Monitoring coverage” was a broad application category, which included reporting overall coverage estimates, providing updated coverage estimates for a given area, or assessing trends in coverage over time. Some authors focused on the quality of coverage (*n =* 19), as well as examining associations between coverage and different variables of interest (*n =* 25), such as mortality outcomes or low birth weight. Of the studies examining associations between coverage and variables of interest, some specifically assessed the impact of an intervention on coverage estimates (*n =* 9) (Table 1).

**FIGURE 5:**
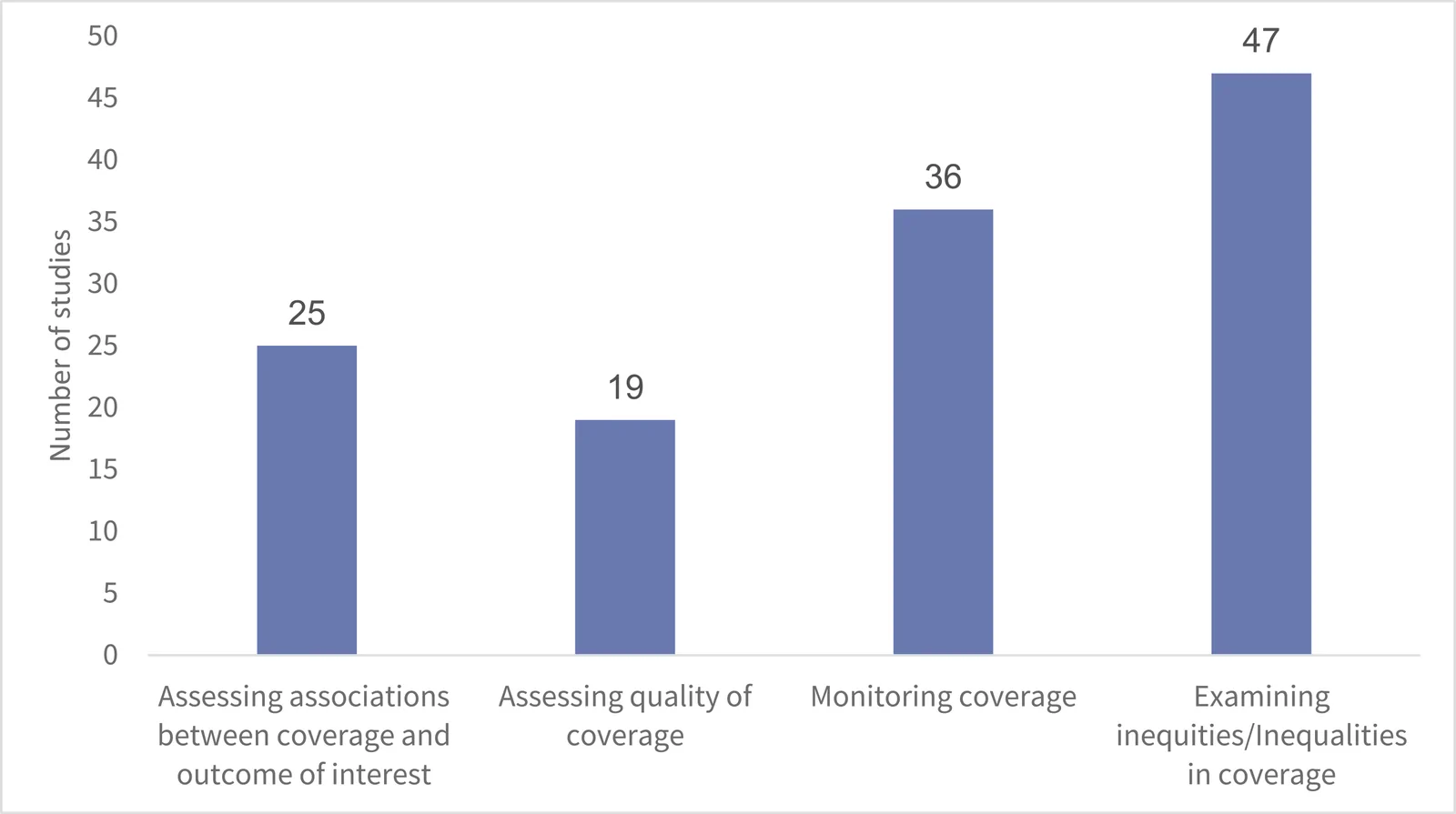
Application of the co-coverage indices across studies. Assessing associations between coverage and outcomes: examining associations between co-coverage and health (mortality or morbidity) or nutrition-related outcomes. Assessing quality of coverage: assessing whether interventions were received at the intended frequency and content (e.g., antenatal care received ≥4 times with required clinical components), not solely whether coverage was met. Monitoring coverage: reporting, updating, or tracking trends in overall coverage estimates for a given area or population. Examining inequities and inequalities in coverage: quantifying inequality in co-coverage and assessing equity-related factors associated with co-coverage.

In addition to understanding how authors calculated and constructed their coverage indices, we extracted methods used to validate such indices. Of the 73 studies, only 18 studies reported formal validation of the co-coverage indices used. Four overarching validation approaches were identified: *1*) agreement methods comparing the index to a reference standard (*n =* 1) [69]; *2*) sensitivity analyses using Spearman rank correlation coefficient (*n =* 2) [118,119] to examine ranked associations, Cronbach’s *α* (*n =* 7) [110,130,121,122] to examine the indices’ internal consistencies, and index correlation (*n =* 5) [14,82,130,69,132] to examine correlations between the index and different indicator weighting approaches; *3*) convergent validity (*n =* 6) [11,52,68,69,73,80] examining the correlation between index estimates and health and nutrition outcomes of interest; and *4*) cross-validation (*n =* 1) [123], which entailed testing index scoring fit and relevance by sequentially including and excluding individual factors in an index (Table 2). We recognize that the Cronbach’s *α* test is designed to assess the internal consistency (reliability) of the indicators included in the index rather than to evaluate validity. Nevertheless, because it is reported in the literature with some frequency, we included it for this review.

**TABLE 2:** Methods used to construct and validate indices

Analytic method	Validation methods^1^						
	*n*	Agreement with reference standards	Sensitivity analysis			Relationship with key outcomes	Other
		Cohen’s κ^1^	Spearman rank coefficient^2^	Index correlation (correlation coefficient)	Cronbach’s *α* reliability coefficient^3^	Convergent validity^4^	Cross-validation^5^
Count	36	0	0	2	1	0	0
Scoring system	2	0	0	0	0	1	0
Simple average	7	0	0	1	0	1	0
Weighted average	36	0	2	2	6	3	1
Geometric mean	3	1	0	1	0	2	0
Principal component analysis	2	1	0	1	0	1	0
Regression weighting	1	1	0	1	0	1	0
Percentage difference	1	0	0	0	0	1	0

1 Cohen’s κ is the agreement between index estimate and reference standard measures.

2 Spearman’s rank coefficient examines ranked correlation.

3 Cronbach’s *α* reliability coefficient examines reliability of a scale/index.

4 Convergent validity refers to the correlation between index estimates and health and nutrition outcomes of interest or potential correlated variables (e.g., stunting among children aged <5 y).

5 Cross-validation refers to the test of index scoring fit and relevance of the indicators (e.g., leave-one-out cross-validation).

## Discussion

Measuring the co-coverage of interventions across sectors is an opportunity to understand how programs and policies converge in a multisectoral approach to improve nutrition. This review found that most studies on this topic were observational, conducted in LMICs in Asia and Africa, and focused on measuring co-coverage at the individual or mother–child dyad level, prioritizing essential curative and preventive interventions related to key life stages. The life stages of focus were primarily pregnancy or infancy, and the continuum of care of focus was the period from preconception to childhood, reflecting investments made in the first 1000 days of life to improve nutrition outcomes [133] and reduce maternal, neonatal, and infant mortality [134]. This finding explains the frequent use of mother–child dyad as the unit of analysis and pregnant and lactating women as the target population.

A gap identified by this review in applying co-coverage indices to assess co-coverage of the full range of multisectoral nutrition interventions is the limited evidence and inclusion of nutrition-sensitive or indirect interventions [135] that address the underlying determinants of malnutrition in co-coverage estimation—a finding noted in other studies [136]. These nutrition-sensitive interventions include provision of social assistance transfers, nutrition-sensitive agriculture interventions, school feeding programs, food fortification interventions, and nutrition education in schools, all of which are often included in multisectoral nutrition policies in LMICs [8,92]. Similarly, co-coverage in a humanitarian context remains sparse. Although our search terms did specify “health” or “nutrition” to ensure that interventions in these areas formed the basis of our review (e.g., not insurance coverage), it was surprising that other sectors (agriculture, social protection, or education) were not well represented, given the multisectoral nature of health and nutritional well-being. Overall, the literature on co-coverage leaned heavily toward health-sector interventions, leaving few studies to guide the development of indices that assess co-coverage or the convergence of multisectoral nutrition integration at the household level. Further, studies that measured the clustering of interventions, focusing on integrated intervention packages to address specific nutrition problems (e.g., anemia control) or multisectoral programs (e.g., multisectoral nutrition initiatives) were scarce.

Another finding of this review is the heterogeneity in methods used to construct co-coverage indices, reflecting both methodological flexibility and a lack of standardization. Across the studies reviewed, 8 main methods for constructing co-coverage indices were identified, with the most common being a simple count of interventions received. Although the simplicity of count-based approaches makes them widely usable, this approach may fail to account for the relative importance or coverage quality of different interventions [137]. Weighted approaches offer more nuance but are subject to bias in how weights are assigned, especially when not based on empirical or policy-driven criteria. Advanced techniques like PCA or regression-weighted methods could offer more rigor but are rarely used, possibly because of complexity or data limitations. This variability in construction methods highlights the need for more explicit justification of methodological choices, transparent documentation of weighting schemes, and consistent reporting practices.

We found few studies that validated the co-coverage indices authors used, with many studies not referencing validation methods for the indices applied. This may be because authors used indices and methodologies applied by other studies. When validation was reported, most studies relied on internal consistency measures like Cronbach’s *α* or correlation coefficients, rather than external benchmarks or outcome-based validations. This validation approach limits confidence in the indices’ reliability and predictive value [138]. The lack of validation is particularly problematic if these measures are to inform policy or track progress over time, especially if seeking to use a method to measure progress between countries [11].

During our search, there were many studies that measured coverage of multiple interventions by combining different data sources and aggregating the data (composite coverage), which reflects the limited availability of data on multiple interventions measured at both the level of the household and individual within the same survey. Furthermore, data to construct co-coverage indices relied heavily on population-based survey data such as the DHS and MICS, which are primarily conducted in LMICs and are a key source of population-level indicators of multisectoral nutrition interventions. The limited evidence from HICs is likely attributable to the origins of the co-coverage method, which was popularized to track child-survival interventions under the Millennium Development Goals—an agenda primarily focused on low-income countries. This finding points to the future challenges in constructing co-coverage indices and tracking co-coverage in the near future, particularly given ongoing disruptions to the DHS program and limited availability of co-coverage data in HIC settings [8,94].

Co-coverage enables policymakers to understand how interventions cluster within a context and to reveal inequities in coverage, especially because intervention targeting is often based on need [14]. Quantifying the magnitude of co-coverage (the proportion of individuals receiving multiple interventions) alongside the comprehensiveness (the average number of recommended interventions received and SD) allows policymakers to gauge the effectiveness of multisectoral program convergence aimed at improving maternal and child health and nutrition outcomes. This review yields a key insight of policy relevance: although several studies conducted multicountry analyses, among those confined to a single country, only 6 co-coverage indices were explicitly grounded in national policies or guidelines. This finding represents a missed opportunity: aligning coverage measurement with the core multisectoral nutrition interventions defined in national or subnational health, nutrition, and agriculture policies and plans. Co-coverage metrics grounded in country-specific national strategies could enhance monitoring, support adaptive learning and resource mobilization, and enable robust accountability mechanisms that are transparent for timely course correction. A transparent approach for interventions prioritized and included in a co-coverage index is also critical. A context-grounded approach can similarly be applied to co-coverage index generation; for example, when identifying indicators using a scoring or weighted average approach, we recommend convening topical and national experts to apply evidence-based and normative methods for prioritizing the scoring and weights assigned to individual interventions. However, this approach can be resource intensive and may limit cross-country comparability, as intervention selection is likely to vary across country contexts.

Future research should apply co-coverage methods to assess clustered interventions within national nutrition-related programs and policies, such as anemia control and multisectoral nutrition strategies. This research need highlights a data gap, necessitating a move beyond coverage measurement of a select set of nutrition interventions to instead capture the full range, enabling a more comprehensive understanding of multisectoral nutrition coverage, identification of unreached populations, and assessment of cross-sectoral coordination and service delivery gaps. Further, validation is essential for any newly developed index, with convergent validity testing and sensitivity analysis representing the minimum standard to confirm the presence of an association between co-coverage and outcomes of interest and understanding how individual indicators shape the overall index. Of note, there is value in measuring co-coverage for monitoring coverage and reach of multiple interventions targetingpopulation, particularly to support advocacy and efforts towards universal coverage [11].

### Strengths and limitations

Strengths of our review include searching a broad range of peer-reviewed evidence and using PRISMA-ScR guidelines to ensure a replicable and rigorous review process. Our search strategy was expansive enough (using proximity operators) to allow for higher variability in how the term co-coverage may have been captured in the literature and allowed for a wide search across disciplines. This is the first review focused on identifying co-coverage methods for potential application.

Limitations of this review include its focus on health- and nutrition-related interventions, which may have excluded relevant studies using alternative terminology in other sectors. The coding of data on intervention selection and prioritization may have been subject to bias, as not all studies reported the rationale for intervention inclusion in the co-coverage index. In such cases, the review team applied judgment based on the examination of the text, which may have introduced miscategorization. We relied on study authors’ own reporting of limitations rather than conducting independent critical appraisal, which may have resulted in some limitations being overlooked. Finally, we had only one study member extract data; however, a subset of extracted data was independently verified by a second reviewer, a third reviewer conducted targeted sensitivity checks, and regular team meetings were held to resolve ambiguities and standardize coding. Although extraction was not performed in duplicate for all studies, these procedures were implemented to maximize accuracy and consistency.

## Conclusion

Co-coverage measurement has advanced as a method to assess integrated efforts to improve maternal and child nutrition, yet it remains largely focused on health-sector interventions. Methods for estimating co-coverage are highly variable and are rarely validated, limiting their comparability and policy relevance. Methods related to index construction sometimes lack reflection of national priorities and transparency related to the weighting of interventions included, which often holds relevance for decision-makers. To enhance their utility, future co-coverage indices should be more inclusive of multisectoral interventions (such as those implemented through the agriculture, water and sanitation, and food industry sectors), aligned with national nutrition strategies, and constructed using transparent, validated methods. Strengthening these elements is essential to effectively apply co-coverage measurement to track progress, guide action, and ensure accountability in multisectoral nutrition efforts.

## Author contributions

The authors’ responsibilities were as follows – SM, SSK: designed the review with support from PN and NP; NP: carried out application of the systematic search with search strategy support from TM, SSK, and SM; NP, SG: carried out screening and coding of studies; SM, PN: resolved conflicts; SG, SM, PN: carried out the analyses; SM, PN, SSK: interpreted the data; SM: drafted the manuscript, supported by SG; and all authors: reviewed the manuscript and read and approved the final manuscript.

## Data availability

The data described in the manuscript will not be made available because no new data were generated.

## Declaration of Generative AI and AI-Assisted Technologies in the Writing Process

The authors declare that no generative AI or AI-assisted technologies were used in the writing of this manuscript.

## Funding

Funding for this study was provided by the Bill & Melinda Gates Foundation (INV-040665), through the DataDENT project, managed byJohns Hopkins University.

## Conflict of interest

The authors report no conflicts of interest.
