# Supplementary material for: Measuring Co-coverage in Global Health and Nutrition Interventions: A Scoping Review of Analytical Approaches
Source: Adv Nutr. 2026 Sep 30;17(11):100742. doi: 10.1016/j.advnut.2026.100742 (PMC13737743; doi:10.1016/j.advnut.2026.100742)
Supplement: Supplementary file 1 [file mmc1.docx]

**Measuring Co-Coverage in Global Health and Nutrition Interventions: A Scoping Review of Analytical Approaches**

**Swetha Manohar**

**SUPPLEMENTARY MATERIAL**

**Supplementary Methods 1: Example search strategy for PubMED**

| **Theme #** | **Search Term (in PubMed)** |
| --- | --- |
| 1 | ("coverage index"[tiab:~3] OR "coverage indices"[tiab:~3] OR "coverage indexes"[tiab:~3] OR "coverage indicator"[tiab:~3] OR "coverage indicators"[tiab:~3] OR "coverage estimate"[tiab:~3] OR "coverage estimates"[tiab:~3] OR "coverage estimated"[tiab:~3] OR "coverage estimation"[tiab:~3] OR "coverage measure"[tiab:~3] OR "coverage measures"[tiab:~3] OR "coverage measurement"[tiab:~3] OR "coverage measurements"[tiab:~3] OR "coverage score"[tiab:~3] OR "coverage scores"[tiab:~3] OR "coverage scoring"[tiab:~3] OR "coverage metric"[tiab:~3] OR "coverage metrics"[tiab:~3] OR "coverage framework"[tiab:~3] OR "coverage frameworks"[tiab:~3] OR "co-coverage index"[tiab:~3] OR "co-coverage indices"[tiab:~3] OR "co-coverage indexes"[tiab:~3] OR "co-coverage indicator"[tiab:~3] OR "co-coverage indicators"[tiab:~3] OR "co-coverage estimate"[tiab:~3] OR "co-coverage estimates"[tiab:~3] OR "co-coverage estimated"[tiab:~3] OR "co-coverage measure"[tiab:~3] OR "co-coverage measures"[tiab:~3] OR "co-coverage measurement"[tiab:~3] OR "co-coverage measurements"[tiab:~3] OR "co-coverage score"[tiab:~3] OR "co-coverage scores"[tiab:~3] OR "co-coverage scoring"[tiab:~3] OR "co-coverage metric"[tiab:~3] OR "co-coverage metrics"[tiab:~3] OR "co-coverage framework"[tiab:~3] OR "co-coverage frameworks"[tiab:~3] OR "co-coverage calculation"[tiab:~3] OR "co-coverage") |
| 2 | (method[ti] OR methods[ti] OR calculate[tiab] OR calculated[tiab] OR calculation[tiab] OR calculations [tiab] OR construct[tiab] OR constructs[tiab] OR construction[tiab] OR constructions[tiab] OR constructing[tiab] OR evaluate[tiab] OR evaluates[tiab] OR evaluated[tiab] OR evaluation[tiab] OR evaluations[tiab] OR weights[tiab] OR weighted[tiab] OR weight[tiab] OR slope[tiab] OR estimate[tiab] OR estimated[tiab] OR estimation[tiab] OR estimations[tiab] OR measure[tiab] OR measures[tiab] OR measured[tiab] OR measurement[tiab] OR measuring[tiab] OR measurements[tiab] OR metric[tiab] OR metrics[tiab] OR index[tiab] OR indexes[tiab] OR indices[tiab]) |
| 3 | ("nutrition intervention"[tiab:~3] OR "nutrition program"[tiab:~3] OR "health intervention"[tiab:~3] OR "health program"[tiab:~3] OR "food intervention"[tiab:~3] OR "food program"[tiab:~3] OR "food fortification"[tiab:~3] OR "mother" OR "mother-child"[tiab:~3] OR "child" OR nutrition OR health) |
| 4 | 1 AND 2 AND 3 |

**Supplementary Methods 2: Operational definitions used in the review to guide the literature search**

| **Term** | **Operational definition** |
| --- | --- |
| **Co-coverage (simplified definition)** | A metric that measures the coverage of multiple recommended nutrition and health interventions received by a target population at the individual or household level (1,2). The operational definition of co-coverage utilized to guide this review was based on seminal literature on the topic. |
| **Composite coverage** | A coverage metric of multiple nutrition and health interventions at a population level (coverage estimates aggregated at the group-level), typically a weighted average and measured using multiple data sources (1). Co-coverage was distinguished from composite coverage which was defined as the coverage measurement of multiple recommended nutrition interventions at a sub-population level (e.g. state, rural/urban, wealth quintile) typically measured using multiple data sources. Composite coverage metrics that met this definition were excluded. |
| **Multi-sectoral nutrition interventions** | Interventions that target the direct or underlying causes of malnutrition. A schema to describe the same can be found in Supplementary Methods 4. |
| **Health interventions** | Interventions that target the major public health problems of a population such as maternal morbidity, child survival, infectious disease, neonatal mortality, and low birth weight. |

**Supplementary Methods 3: Systematic scoping review inclusion/exclusion criteria**

| **Inclusion Criteria** | **Exclusion Criteria** |
| --- | --- |
| **Population** | |
| - General population (no restrictions placed on specific clinical, demographic, or geographic characteristics) - Levels included: measurements at the population-, household- or individual- (including dyad) level | - Animal studies - Levels excluded: measurement at the sub-population level (e.g. state, rural/urban, wealth quintile) |
| **Intervention/ Exposure** | |
| - Health and/or nutrition intervention coverage with assessment of co-coverage (as exposure or outcome) - Assessment of co-coverage (as exposure or outcome) - Composite coverage from a single data source* | - Health and/or nutrition intervention coverage assessed but co-coverage NOT assessed. - Focus on vaccine coverage ONLY (multiple doses/vaccines) - Composite coverage indices using multiple data sources, such as Composite Coverage Index, UHC Service Coverage Index, Coverage Gap Index. - Studies on the country or community level (aggregate) |
| **Comparator / Context** | |
| - Studies in any country (high income, upper middle income, low-middle income). |  |
| **Study Characteristics** | |
| - Original peer-reviewed studies including protocols (must include a clear description of construction and application of co-coverage index), book chapters, technical reports, dissertation/ thesis - Use quantitative methods - Mixed methods studies - Experimental and Observational studies. - Household & demographic surveys (study surveys, Demographic and Health Surveys, Multiple Indicator Cluster Surveys, national health surveys, etc.) - Multiple rounds of the same demographic/household surveys - Research records, facility records/ administrative data, direct observation - Data primarily from a single source (unless individual data linkage is used) - Published on or after 1990 - Full-text in English language available | - Entire books, commentaries, conferences abstracts, book review, and letters, editorials, op-eds, - Reviews (systematic, scoping review, meta-analysis) - Use qualitative methods only - Studies combining data from multiple sources (unlinked at the individual level) to create aggregate coverage measures - Published before 1990 - Full-text in English language not available |

*The terms ‘composite coverage’ and ‘co-coverage’ are sometimes used interchangeably because co-coverage indices can be a composite measure (i.e. a composite indicator is a summary indicator that combines different data to summarize coverage). There are distinctions made in the literature for coverage measurement between a composite and co-coverage indicator. The operational definitions (described in Supplementary Methods 2) guided application of the inclusion and exclusion criteria for this review.

**Supplementary Methods 4**: **List of key multisectoral nutrition interventions over the life-course at the individual and household levels^§^**

| **Individual level interventions** | | | | | **Household level interventions*** |
| --- | --- | --- | --- | --- | --- |
| **Adolescence** | **Preconception** | **Pregnancy** | **Delivery and lactation** | **Early Childhood** |  |
| - Daily or intermittent iron and folic acid (IFA)* supplementation - Preventive deworming* - Food supplementation - Counseling/ education on healthy diets - Cash/food/in-kind social protection transfer | - Daily or intermittent IFA supplementation* - Preventive - deworming* - Contraception - Salt fortification (iodine supplementation) - Cash/food/in-kind social protection transfer | - Antenatal care (ANC) screening by a trained provider - ANC screening in 1st trimester by a trained provider - At least 4 ANC visits - Energy and protein dietary supplementation* - Daily or intermittent IFA supplementation* - Vitamin A supplementation* - Calcium supplementation* - Iron-containing micronutrient powder (MNP) supplementation* - Preventive deworming* - Tetanus toxoid vaccination - Nutritional counseling on healthy diet* - Weight monitoring - Advice about weight after weighing - Advice on consuming calcium - Advice on consuming IFA - Advice on consuming additional food - Advice on birth preparedness - Advice on exclusive breastfeeding - Cash/food/in-kind social protection transfer | - Institutional birth - Skilled birth attendant - Optimal timing of (delayed) cord clamping* - Assessment of birth weight - Support for exclusive breastfeeding (EBF) and skin-to-skin contact* - Optimal feeding of low birth weight (LBW) infants* - Counsel mothers of LBW infants on kangaroo mother care (KMC)* - Postnatal care for babies - Postnatal care for women - Breastfeeding counseling* - Daily or intermittent IFA supplementation* - Food supplementation for malnourished lactating women - Cash/food/in-kind social protection transfer | - Breastfeeding counseling* - Counseling on appropriate complementary feeding* - Food supplementation for complementary feeding* - Iron- containing MNP* - Daily or intermittent IFA supplementation*^10^ - Zinc during diarrhea* - Oral rehydration salt (ORS) during diarrhea* - Vitamin A supplementation* - Preventive deworming* - Growth monitoring* - Counseling on nutritional status* - Identification of severely or moderately underweight* - Inpatient management of severe acute malnutrition (SAM)* - Outpatient management of SAM* - Management of moderate acute malnutrition (MAM)* - Immunization - Cash/food/in-kind social protection transfer | - Large scale food fortification - Nutrition-sensitive agriculture - Water, sanitation and hygiene - Cash/food/in-kind social protection transfer |
| **^§^** Adapted with perfmission from Neupane S, Jangid M, Scott SP, et al. Availability of national policies, programmes, and survey-based coverage data to track nutrition interventions in South Asia. Matern Child Nutr2024;20:e13555. doi:10.1111/mcn.13555 pmid:37592411  *Recommended WHO under Essential Nutrition Actions Mainstreaming Nutrition through the Life-Course.  * Household-level interventions include agriculture, social protection, or nutrition-sensitive programs | | | | |  |

**Supplementary Methods 5: Coding structure for data extraction for health and nutrition intervention coverage studies**

| Code Domains | Codes | Definition |
| --- | --- | --- |
| *Title* | Free text | Title of paper/report |
| *First Author* | Free text | First author of paper |
| *First Author’s Affiliation* | Free text | Organization/ institution/ university, etc. the first author is affiliated with |
| *Publication Year* | Free text | Year that the paper/report was published |
| *Journal/Place of Publication* | Free text | Name of journal where article was published (e.g. Maternal & Child Nutrition) or institution of publication for reports (e.g. Save The Children Resource Centre) |
| *Article Type* | Select one   - Research Article - Protocol - Technical Report - Other (Working papers, Pre-print) | Categorization of the type of evidence as research article (peer-reviewed study), protocol (study protocol), technical report (non-peer reviewed report) and other (working papers, pre-print). |
| *Country/Countries* | Free text | Name of country or countries in which the data analyzed was collected. |
| *Region* | Select multiple   - East Asia and Pacific - Europe and Central Asia - Latin America & the Caribbean - Middle East & North Africa - North America - South Asia - Sub-Saharan Africa - N/A | Where individual countries and regions fall; use region names based on [World Bank country classifications](https://datahelpdesk.worldbank.org/knowledgebase/articles/906519-world-bank-country-and-lending-groups). |
| *Income Context* | Select multiple   - Low Income & Lower-middle Income - Upper-middle Income - High Income | Income context of country or countries of which data analyzed come from, according to the [World Bank 2024-2025 Country Classifications by Income Level](https://blogs.worldbank.org/en/opendata/world-bank-country-classifications-by-income-level-for-2024-2025#:~:text=The%20World%20Bank%20Group%20assigns%20the%20world%E2%80%99s%20economies,GNI%20per%20capita%20of%20the%20previous%20calendar%20year.). |
| *Rural/Urban Context* | Select multiple   - National - Rural - Urban - Other (Peri-urban, slum, etc.) - N/A | Describing the rural/urban context as described in the setting. N/A refers to if the context is not defined and not reported. If MICS or DHS, coded as a national dataset. |
| *Humanitarian Context* | Free text | Any description of a humanitarian/disaster/conflict area. |
| *Type of Research* | Select one   - Observational - Experimental | Specify whether the study was observational (e.g. cross-sectional epidemiological studies like the DHS with no interventions or assessing associations between an outcome and exposure) or experimental (e.g. studies using experimental methods like impact evaluations using a randomization or quasi-experimental methods) |
| *Research Method* | Select one   - Quantitative - Mixed Methods | Designate the methodological approach the article adopted. |
| *Study Aim* | Free text | Copy the aim of the overall study as defined by the authors |
| *Sample Size* | Free text | Sample size stated by authors containing as many details concerning sample size as provided including different sample sizes for different analyses and stratifications.  If there are multiple indices, specify unit – ex. women aged 15-49, children under 5, etc. |
| *Study Outcome(s)* | Free text | Describe outcomes examined (e.g. wasting, stunting, Minimum Dietary Diversity score, etc.). |
| *Name of Coverage Index* | Free text | Identify specific name(s) the authors used for coverage index used. |
| *Terms Used to Describe Coverage Index* | Select one   - Uses “co-coverage” - Uses “composite coverage” - Uses “co-coverage” and “composite coverage” interchangeably for the same index - Uses “co-coverage” and “composite coverage” separately for different indices in the paper - Neither “co-coverage” nor “composite coverage” used | Designate whether authors used terms “co-coverage” and/or “composite coverage” to gain insight into how these specific terms are used in the literature |
| *Category of Interventions Included in Index* | Select one:   - Health and Nutrition-specific & RMNCH - Nutrition-sensitive (non-health sector) - Both nutrition-sensitive & nutrition-specific interventions included - Other | Designate what category of interventions and services are included in the coverage index, categorizing micronutrient supplementation and healthcare services as nutrition-specific and RMNCH, respectively and access to drinking water as nutrition-sensitive.  Capturing the text they use to describe the categories (selecting the text they use to describe during extraction).  ‘Other’ would include interventions that do not fall under health and nutrition-specific or sensitive interventions |
| *No. of Interventions in Coverage Index* | Free text | Designate the specific number and type of interventions contained in the coverage index. If multiple different co-coverage/composite indices were calculated in the paper, designate the number of interventions included in the calculation of each. |
| *Specific Interventions in Coverage Index* | Select multiple  *Household level interventions*  *Agriculture interventions*   - NSA - home gardens - NSA - improved seed & nutrition counseling - NSA - livestock provision - NSA - hydroponic ponds - NSA - provision of seeds for indigenous foods   Nutrition interventions   - Universal salt iodization - Vitamin A fortified oil - Vitamins A, B1, B12, Fe, FA, zinc in rice - Vitamins A and D of milk - Iron, folic acid, and Vitamin B12 of wheat flour - Iron, folic acid, and Vitamin B12 in fortified of wheat flour - Zinc, Vitamins A, B1, B2, B3, & B6 fortified wheat flour   WASH interventions   - Improved sanitation - Availability of drinking water - Handwashing facilities with soap & water   *Individual-level interventions*  Health interventions   - Optimal timing of umbilical cord clamping - Paid maternity leave - Kangaroo care for low birth weight - Identify infants < 6m with severe acute malnutrition - Inpatient management of infants < 6m with severe acute malnutrition - Preventive deworming - child - Zinc during diarrhea - Immunization - Anemia assessment - Anemia treatment (iron supplementation) - Antenatal care screening by a trained provider - Tetanus toxoid vaccination - Institutional delivery - Presence of skilled birth attendant - Postnatal care for women - Postnatal care for babies - Contraception - Preventative deworming - women - Other: free text   Nutrition interventions   - Education on healthy diets - Early breastfeeding support & counseling - facility & community - Support for early breastfeeding and skin-to-skin contact - Prevent breastmilk substitutes & formula distribution at hospitals - Continued breastfeeding counseling - Optimal feeding of low birth weight and very low birth weight - Outpatient management of infants < 6m with severe acute malnutrition & moderate acute malnutrition - Vitamin A supplementation - Nutrition counseling on complementary feeding - Weight and height/length assessment - Nutrition counselling for children - Iron-containing micronutrient powder for point-of-use fortification of foods - Daily iron supplementation for infants and young children aged 6-23 m - Intermittent iron & iron-folic acid supplementation for non-pregnant women - Iron & iron-folic acid supplementation for pregnant women - Daily Fe supplementation for women - School feeding - Nutritional counseling on healthy diet to reduce the risk of low birth weight - Nutrition counseling - general - Energy and protein dietary supplements in undernourished populations - Vitamin A supplementation for pregnant women - Calcium supplementation   Nutrition-sensitive social protection   - NSSP - cash transfer - NSSP - cash transfer - Assessment of blood pressure - Blood glucose testing - Weight monitoring - Advice on weight monitoring | Select the specific interventions and services included in the calculation of the coverage index (e.g. IFA supplementation, breastfeeding counselling, etc.). Training and discussions had on different intervention types. During analysis, further re-categorization conducted to aggregate data. |
| *Selection/Prioritization of Interventions in Coverage Index* | Free text | Describe any justification, rationale, and reasons the authors provide for selecting and including the different interventions and services included in their coverage indices (ex. expert opinion, specific index has been used elsewhere, global recommendations, etc.). |
| *Type of Data Used for Index* | Select multiple   - Survey/other study type - Records/Administrative Data - Direct Observation - Other | Designate the type of data used to calculate the coverage index. Survey data includes DHS, MICS, other national surveys like epidemiological nationally-representative surveys (e.g. national nutrition surveys), and other study types includes data from independent studies typically collecting cross-sectional survey data but also data from included experimental trials. Records/administrative data may refer to health center records, ANC cards, etc. Other included studies that reported multiple data sources. |
| *Data Source for Coverage Index* | Select multiple   - DHS - MICS - Other National Survey - Study Survey - Health Center Records - Antenatal Care Card - Other | Designating the specific source of data used for calculation of the authors’ coverage index. Other study surveys included other surveys like nationally-representative surveys (e.g. national nutrition surveys) or other epidemiological surveys. If the authors calculated multiple different co-coverage indicators with different data sources, select all data sources. Antenatal care cards are cards issued to women to keep track of their visits. |
| *Multiple Rounds of Data* | Free text | Description of data sources across multiple rounds/years from the same country or region |
| *Unit of Analysis* | Select multiple   - Individual Level - Mother-Child Dyad Level - Household Level - Other (e.g. community) | Identify the unit of analysis used for calculating the co-coverage/composite coverage index (not necessarily the broader analysis).  Individual level: Intervention coverage measured at individual level for a specific target population (e.g.: children under five years of age)  Mother-child dyad level: Intervention coverage measured for shared interventions received for women during the antenatal and postnatal period. This included household interventions like access to safe water because this was coverage that was shared across mother and child.  Household level: Intervention coverage measured at the household level only, for e.g., handwashing access for the household.  Composite indicators or those that combined more than one source of data were coded as ‘other’ too but, because we only included articles that linked data at the individual level, ‘other’ was not a mutually exclusive category. |
| *Population of Analysis (Population level)*  Select multiple | Select multiple   - Children under 5 years - Women 15-49 years - Mother-children under 5 pair - Children under 2 years - Mother-children under 2 years pairs - Adolescent girls - Pregnant & lactating women - Other | Identify the specific population authors are analyzing and using data from to calculate their co-coverage/composite coverage measure.  If authors calculated multiple different co-coverage measures among different populations of interest, select all different populations of analysis.  Select ‘pregnant and lactating women’ if the study describes recruiting pregnant (e.g.: da Fonesca 2014) or postnatal women (e.g.: Prinja 2017) into the study but does not specify age criteria for the study. Select ‘women 15-49 years’ if the study specifies this age group or if women’s data from the DHS and MICS or DHS national survey equivalents are used (e.g.: National Family Health Survey in India or the National Health Service Survey in China) since women 15-49 years is the target population for these surveys and related women indicators. There will likely be substantial overlap between pregnant & lactating women and women 15-49 years of age. |
| *Calculation Details* | Free text | Extract the authors’ description of how they calculated their co-coverage/composite coverage index. Include any formulas used, descriptions of weighting, etc. Include as many details as provided in the paper and review at supplementary files if relevant. |
| *Covariates Used* | Free text | Extract and describe any covariates and control variables used by authors in different analyses involving the co-coverage/composite coverage index (ex. age, sex, district, etc.). |
| *Statistical Analyses Conducted* | Free text | Describe any statistical analyses that the authors conducted with the co-coverage/composite coverage measure (ex. any regression models, prevalence/descriptive analyses, time trends, etc.). Include as many details as provided in the paper and look at supplementary files if relevant. |
| *Validation Methods of Index* | Free text | Extract and describe any validation methods used for the coverage index |
| *Application of Coverage Index* | Select multiple   - Monitoring of coverage - Assessing quality of coverage - Examining inequities and inequalities in coverage - Assessing associations between coverage and outcome of interest - Other: free text - Not specified | This is a broad code with different ways in which the authors use and suggest using the coverage index – may be found in the discussion section; may be overlap with aim.  Monitoring coverage: Reporting, updating, or tracking trends in overall coverage estimates for a given area or population.  Quality of coverage: Assessing whether interventions were received at the intended frequency and content (e.g., antenatal care received at least four times with required clinical components), not solely whether coverage was met.  Examining inequities and inequalities in coverage: Quantifying inequality in co-coverage and assessing equity-related factors associated with co-coverage.  Assessing associations between coverage and outcomes: Examining associations between co-coverage and health (mortality or morbidity) or nutrition-related outcomes. |
| *Research & Methodological Recommendations* | Free text | Include any recommendations that the authors describe for future research, for data collection, for calculating co-coverage indices, etc. – may be found towards the end of the paper in the discussion or conclusion, but may also find some recommendations in the methods section or elsewhere (this code does not include program or policy recommendations – e.g., improving access to health services, but rather, is focused on methodological and research recommendations). |
| *Limitations* | Free text | Include any limitations that the authors describe in their study and analysis (e.g. small sample sizes, etc.); typically, in a limitations section towards the end of the paper |
| *Additional Info* | Free text | Any additional relevant information from the article that is not contained elsewhere in the data extraction, but that may be useful moving forward |
| *Questions* | Free text | Any questions the individual reviewing the article has about the study – for later discussion with the team |
| Research question | | Related codes |
| #1. What is the evidence on selection and prioritization of interventions to use in co-coverage analysis? E.g., global recommendations/evidence-based, expert consultations. | | - Category of Interventions Included in Index - Type of Interventions in Index - Specific Interventions in Coverage Index - Reasons for Selection/Prioritization of Interventions in Coverage Index |
| #2. What are the existing analytical methodologies used for assessing co-coverage? What co-coverage metrics and their construction are described? What is the unit of analysis used (e.g., individual, dyad, household, other administrative unit)? What are considerations and trade-offs (weighting items, data-driven approaches like PCA)? | | - Type of Research - Research Method - No. of Interventions in Index - Type of Data Used for Index - Data Source for Coverage Index - Unit of Analysis - Population of Analysis - Calculation Details - Statistical Analyses Conducted - Covariates Used - Validation Methods of Index - Research & Methodological Recommendations - Limitations |
| #3. How have the methodologies been applied across different contexts and settings? This includes examining the breadth of applications and any variations or adaptations of the methodologies to different contexts. How have these methodologies been validated? | | - Country/Countries - Region - Income Context - Rural/Urban Context - Humanitarian Context - Study Aim - Study Outcome(s) - Application of Coverage Index |

DHS: Demographic Health Surveys, MICS: Multiple Indicator Cluster Surveys, NSA: Nutrition-sensitive agriculture, NSSP: Nutrition-sensitive social protection, RMNCH: Reproductive, maternal, newborn and child health,

**Supplementary Table 1:** Summary of study characteristics included in the review*

| **Domain** | **Characteristic** | | **Frequency (n)** |
| --- | --- | --- | --- |
| **General** | Evidence type | Research article | 72 |
|  |  | Protocol | 1 |
| **Country context** | Single vs. multiple | Single country | 49 |
|  |  | Multiple countries | 24 |
|  | Country income context | Low-middle income | 67 |
|  |  | Upper-middle income | 24 |
|  |  | High income | 8 |
| **Time trends** | Publication year | 2005-2009 | 2 |
|  |  | 2010-2014 | 8 |
|  |  | 2015-2019 | 22 |
|  |  | 2020-2024 | 41 |
| **Methods** | Study design | Observational | 66 |
|  |  | Experimental/ Quasi-experimental | 7 |
|  | Research method | Quantitative | 72 |
|  |  | Mixed-Methods | 1 |
|  | Data representativeness | National | 50 |
|  |  | Urban | 8 |
|  |  | Peri-Urban | 1 |
|  |  | Rural | 17 |
|  |  | Unspecified | 5 |
|  | Data types | Survey/other primary data  (data from other study type) | 68 |
|  |  | Records/ administrative data | 2 |
|  |  | Multiple data types^†^ | 3 |
|  | Application | Monitoring coverage  Coverage equity  Quality of coverage  Association between coverage and outcome of interest (nutritional status, mortality) | 36  47  19  25 |
|  | Data sources - coverage index computation | Demographic Health Surveys | 41 |
|  |  | Multiple Indicator Cluster Surveys | 16 |
|  |  | Other national survey | 16 |
|  |  | Sub-national survey/ other studies^‡^ | 16 |
|  |  | Health center records | 1 |
|  |  | Personal health records (ANC card) | 2 |
|  |  | Census | 1 |
|  |  | Direct observation | 1 |
|  | Unit of analysis | Mother-child dyad | 28 |
|  |  | Individual | 49 |
|  |  | Household | 1 |
|  |  | Other  (individual data used to measure CCI & CGI) | 30 |

ANC card: Antenatal card, CCI: Composite Coverage Index, CGI: Coverage Gap Index

* Summary statistics presented are not additive to the total number of articles included, as many articles covered multiple codes.

^†^ Multiple data types included studies that both included household surveys combined with direct observation of interventions or health records (ANC card) or linkages to other data sources.

^‡^ Sub-national/other studies: This includes subnational surveys and non-survey research studies

**Supplementary Table 2: List of studies included in the review**

| **Author/year** | **Title (reference)** |
| --- | --- |
| Victora 2005 | Co-coverage of preventive interventions and implications for child-survival strategies: evidence from national surveys (3) |
| Boerma 2008 | Mind the gap: equity and trends in coverage of maternal, newborn, and child health services in 54 Countdown countries (4) |
| Barros 2012 | Equity in maternal, newborn, and child health interventions in Countdown to 2015: a retrospective review of survey data from 54 countries (5) |
| Rai 2012 | District level coverage gap in Maternal, Newborn and Child Health care services in India (6) |
| Kumar 2013 | Coverage gap in maternal and child health services in India: assessing trends and regional deprivation during 1992-2006 (7) |
| Singh 2013 | Equity in maternal, newborn, and child health care coverage in India (8) |
| Victora 2013 | How within-country inequalities and co-coverage may affect LiST estimates of lives saved by scaling up interventions (9) |
| daFonseca 2014 | Adequacy of antenatal care and its relationship with low birth weight in Botucatu, Sao Paulo, Brazil: a case-control study (10) |
| Hodgins 2014 | The quality-coverage gap in antenatal care: toward better measurement of effective coverage (11) |
| Miller 2014 | Coverage and equitability of interventions to prevent child mortality in rural Jimma and West Hararghe Zones, Oromia Region, Ethiopia (12) |
| Marchant 2015 | Adding Content to Contacts: Measurement of High-Quality Contacts for Maternal and Newborn Health in Ethiopia, North East Nigeria, and Uttar Pradesh, India (13) |
| Restrepo-Méndez 2015 | Progress in reducing inequalities in reproductive, maternal, newborn, and child health in Latin America and the Caribbean: an unfinished agenda (14) |
| Akseer 2016 | Coverage and inequalities in maternal and child health interventions in Afghanistan (15) |
| Wehrmeister 2016 | Summary indices for monitoring universal coverage in maternal and child health care (1) |
| Carvajal-Aguirre 2017 | Gap between contact and content in maternal and newborn care: An analysis of data from 20 countries in sub-Saharan Africa (16) |
| Koroma 2017 | The quality of free antenatal and delivery services in Northern Sierra Leone (17) |
| McDougal 2017 | Making the continuum of care work for mothers and infants: Does gender equity matter? Findings from a quasi-experimental study in Bihar, India (18) |
| Oliveira-Ciabati 2017 | PRENACEL - a mHealth messaging system to complement antenatal care: a cluster randomized trial (19) |
| Prinja 2017 | A composite indicator to measure universal health care coverage in India: way forward for post-2015 health system performance monitoring framework (20) |
| Roche 2017 | A long way to go - Estimates of combined water, sanitation and hygiene coverage for 25 sub-Saharan African countries (21) |
| Sanoussi 2017 | Measurement and analysis of inequality of opportunity in access of maternal and child health care in Togo (22) |
| Servan-Mori 2017 | Use of Performance Metrics for the Measurement of Universal Coverage for Maternal Care in Mexico (23) |
| Triyana 2017 | The effects of a household conditional cash transfer programme on coverage and quality of antenatal care: a secondary analysis of Indonesia's pilot programme (24) |
| Victora 2017 | The contribution of poor and rural populations to national trends in reproductive, maternal, newborn, and child health coverage: analyses of cross-sectional surveys from 64 countries (25) |
| Benova 2018 | Two decades of antenatal and delivery care in Uganda: a cross-sectional study using Demographic and Health Surveys (26) |
| Garchitorena 2018 | Early changes in intervention coverage and mortality rates following the implementation of an integrated health system intervention in Madagascar (27) |
| Keats 2018 | Assessment of Inequalities in Coverage of Essential Reproductive, Maternal, Newborn, Child, and Adolescent Health Interventions in Kenya (28) |
| Thomson 2018 | Impact of a health system strengthening intervention on maternal and child health outputs and outcomes in rural Rwanda 2005-2010 (29) |
| Akseer 2019 | Association of Exposure to Civil Conflict With Maternal Resilience and Maternal and Child Health and Health System Performance in Afghanistan (30) |
| Modi 2019 | mHealth intervention "ImTeCHO" to improve delivery of maternal, neonatal, and childcare services-A cluster-randomized trial in tribal areas of Gujarat, India (31) |
| Sakuma 2019 | Determinants of continuum of care for maternal, newborn, and child health services in rural Khammouane, Lao PDR (32) |
| Zhang 2019 | Trends and projections of universal health coverage indicators in Ghana, 1995-2030: A national and subnational study (33) |
| Barros 2020 | Are the poorest poor being left behind? Estimating global inequalities in reproductive, maternal, newborn and child health (34) |
| Bouilly 2020 | Maternal and child health inequalities among migrants: the case of Haiti and the Dominican Republic (35) |
| Carvajal 2020 | Basic maternal health care coverage among adolescents in 22 sub-Saharan African countries with high adolescent birth rate (36) |
| Garchitorena 2020 | District-level health system strengthening for universal health coverage: evidence from a longitudinal cohort study in rural Madagascar, 2014-2018 (37) |
| Hategeka 2020 | Temporal trends in coverage, quality and equity of maternal and child health services in Rwanda, 2000-2015 (38) |
| Hermann 2020 | Mothers' Preventive Health Care Practices and Children's Survival in Burkina Faso: Findings from Repeated Cross-sectional Household Surveys (39) |
| Odhiambo 2019 | Measuring health system resilience in a highly fragile nation during protracted conflict: South Sudan 2011-15 (40) |
| Oh 2020 | Factors associated with the continuum of care for maternal, newborn and child health in The Gambia: a cross-sectional study using Demographic and Health Survey 2013 (41) |
| Panda 2020 | District level inequality in reproductive, maternal, neonatal and child health coverage in India (42) |
| Ramesh 2020 | The contribution of district prioritization on maternal and newborn health interventions coverage in rural India (43) |
| Thapa 2020 | Equity and Coverage in the Continuum of Reproductive, Maternal, Newborn and Child Health Services in Nepal-Projecting the Estimates on Death Averted Using the LiST Tool (44) |
| Wang 2020 | Equity in Essential Maternal, Newborn, and Child Health Interventions in Northeastern China, 2008 to 2018 (45) |
| Wehrmeister 2020 | Measuring universal health coverage in reproductive, maternal, newborn and child health: An update of the composite coverage index (46) |
| Wehrmeister 2020 | Wealth-related inequalities in the coverage of reproductive, maternal, newborn and child health interventions in 36 countries in the African Region (47) |
| Anindya 2021 | Socioeconomic inequalities in effective service coverage for reproductive, maternal, newborn, and child health: a comparative analysis of 39 low-income and middle-income countries (48) |
| Arroyave 2021 | Inequalities in antenatal care coverage and quality: an analysis from 63 low and middle-income countries using the ANCq content-qualified coverage indicator (49) |
| Gandhi 2021 | Level of inequality and the role of governance indicators in the coverage of reproductive maternal and child healthcare services: Findings from India (50) |
| Kothavale 2021 | Level of completion along continuum of care for maternal, newborn and child health services and factors associated with it among women in India: a population-based cross-sectional study (51) |
| Peven 2021 | Household Survey Measurement of Newborn Postnatal Care: Coverage, Quality Gaps, and Internal Inconsistencies in Responses (52) |
| Peven 2021 | Equity in newborn care, evidence from national surveys in low- and middle-income countries (53) |
| Sanhueza 2021 | SDG3-related inequalities in women's, children's and adolescents' health: an SDG monitoring baseline for Latin America and the Caribbean using national cross-sectional surveys (54) |
| Arsyi 2022 | Antenatal Care Services and Incidence of Low Birth Weight: A Comparison of Demographic and Health Surveys in 4 ASEAN Countries (55) |
| Baye 2022 | Co-coverage of reproductive, maternal, newborn and child health interventions shows wide inequalities and is associated with child nutritional outcomes in Ethiopia (2005-2019) (56) |
| Khatri 2022 | High coverage but low quality of maternal and newborn health services in the coverage cascade: who is benefitted and left behind in accessing better quality health services in Nepal? (57) |
| Kim 2022 | Measuring effective coverage of maternal and child health services in Cambodia: a retrospective analysis of Demographic and Health Surveys from 2005 to 2014 (58) |
| Lindberg 2022 | The Extent of Universal Health Coverage for Maternal Health Services in Eastern Uganda: A Cross Sectional Study (59) |
| Nguyen 2022 | Unequal coverage of nutrition and health interventions for women and children in seven countries (60) |
| Worku 2022 | Maternal Service Coverage and Its Relationship To Health Information System Performance: A Linked Facility and Population-Based Survey in Ethiopia (61) |
| Zhou 2022 | Universal health coverage in China: a serial national cross-sectional study of surveys from 2003 to 2018 (62) |
| Blanchard 2023 | Going deeper with health equity measurement: how much more can surveys reveal about inequalities in health intervention coverage and mortality in Zambia? (63) |
| Cisse 2023 | Evaluation of maternal and childcare continuum in Guinea: a secondary analysis of two demographic and health surveys using the composite coverage index (CCI) (64) |
| Das 2023 | Community Mobilization and Community Incentivization (CoMIC) Strategy for Child Health in a Rural Setting of Pakistan: Study Protocol for a Randomized Controlled Trial (65) |
| Gebremedhin 2023 | Determinants of continuum of care for maternal, newborn, and child health services in Ethiopia: Analysis of the modified composite coverage index using a quantile regression approach (66) |
| Gebremedhin 2023 | Effective coverage of newborn postnatal care in Ethiopia: Measuring inequality and spatial distribution of quality-adjusted coverage (67) |
| Girotra 2023 | Utilization and determinants of adequate quality antenatal care services in India: evidence from the National Family Health Survey (NFHS-5) (2019-21) (68) |
| Methun 2023 | Inequalities in adequate maternal healthcare opportunities: evidence from Bangladesh Demographic and Health Survey 2017-2018 (69) |
| Tauhidi 2023 | Measuring Coverage of Essential Maternal Postnatal Care Services in the Squatter Settlements of Islamabad Capital Territory in Pakistan (70) |
| Vidaletti 2023 | Time trends in ethnic inequalities in child health and nutrition: analysis of 59 low and middle-income countries (71) |
| Nguyen 2024 | Identifying measures for coverage of nutrition-sensitive social protection programs: Learnings from India (72) |
| S 2024 | Wealth and education-related inequalities in the utilisation of reproductive, maternal, newborn, and child health interventions within scheduled tribes in India: an analysis of Odisha and Jharkhand (73) |
| Shibre 2024 | Explaining changes in educational disparities in competent maternal health care services in urban and rural areas in Ethiopia (74) |

**Supplementary Figure 1: Target population for co-coverage indices**

|  | Number of studies |
| --- | --- |
| Infants | 11 |
| Children under 2y | 39 |
| Children under 5y | 36 |
| Women 15-49y | 61 |
| Pregnant and lactating women | 70 |
| Other* | 4 |

*Includes adult inpatient population, population with non-communicable diseases/ other diseases, adolescents, households

**Supplementary Table 3**: **Analytical methodologies used for co-coverage assessments**

| **Analytic approach** | **Estimation of index/ formula**  **(illustrative example)** | **Metric**  **(illustrative example)** | **Studies** | **Additional Notes** |
| --- | --- | --- | --- | --- |
| ***Count*** | **Co-coverage** S**core**= BCG +DPT +MSL +Tetanus + VitA + ANC + SBA + safe water  (55) | - Score: 0-8 - Cut-offs to make binary category: - Binary indicator of 1 for 6+ interventions and 0 for <6 interventions. - Cut off to make ordinal categories: - High coverage: ≥ 6 interventions - Medium coverage: 3-5 interventions - Low coverage: 0-2 int. (55) | (1, 3, 15, 50, 55, 56, 73) | Restricted to children 6-23 months in mother-child pairs (56).  One paper calculated a score of 0-7 due to missing tetanus vaccination data in some surveys. |
|  | **Co-coverage Score** = ANC+SBA+DPT+WAT+VTA+ITN | - Score: 0-6 | (9) |  |
|  | **Co-coverage Score** = BCG +OPV-3 + Penta-3 +MSL +VitA +ITN or IRS +Safe water +Improved sanitation | - Score: 0-8 - Cutoff to make binary indicator for co-coverage: ≥4 interventions or none | (12) | Restricted the co-coverage calculation to children 12-35 months old to maximize sample size of included interventions. |
|  | **Co-coverage Score** = ANCS + Tetanus + SBA + VitA + BCG + DPT3 + MSL + improved drinking water + ITN | - Score: 0-9 - Cutoff to make binary category: co-coverage if 6+ interventions received and <6 interventions. | (28) |  |
|  | **Continuum of Care Co-Coverage** = ANC4 + SBA + clean cord care + skin-to-skin care + delayed first bath + early breastfeeding + PNC home visit + exclusive breastfeeding + postpartum contraception | - Score: 0-9 | (18) |  |
|  | **Co-coverage of health interventions** = ANC4 + 2 tetanus + SBA + Child Vit A + BCG + MSL + DPT + improved drinking water | - Score: 0-8 - Cutoff to make binary category: ≥4 interventions or none | (60) |  |
|  | **Co-coverage of nutrition interventions** = household consumption of iodized salt + consuming iron supplements for at least 100 days during pregnancy + receiving deworming tablets during pregnancy + early initiation of breastfeeding + child weight measurement at birth + child iron supplementation + child VitA + child deworming | - Score generated; range: 0-8 - Computed proportion of mother-child pairs received ≥4 interventions | (60) |  |
|  | **Co-Coverage of ANC interventions** = Among women with ANC (either at least 1 ANC or 4+ ANC), receipt of the following 8 interventions: urine test + blood pressure taken + blood sample + iron supplementation + tetanus protection + counselled on pregnancy complications + tested for HIV and received results + IPTP | - Count of 8 interventions - Percent of women with ANC (either 1+ or 4+ ANC) receiving all 8 interventions - Used as binary outcome in logistic regression | (16) |  |
|  | **Co-Coverage of PNC interventions** = Among women with a SBA, newborn receipt of the following 7 interventions: newborn weighed at birth + early initiation of breastfeeding + no pre-lacteal feed during first 3 days of life + BCG vaccination + polio vaccination at birth + PNC for newborn within 2 days + PNC for mother within 2 days | - Count of 7 interventions - Percent of women with SBA whose newborn received all 7 interventions - Used as binary outcome in logistic regression | (16) |  |
|  | **Co-coverage of the basic package of maternal health care** = 4+ ANC + having received 4 ANC content intervention (BP measured, blood and urine sample taken, received tetanus toxoid injection) + having had an SBA at birth + initiating breastfeeding within 1 hour | - Count of 4 intervention - Binary outcome used (received all 4 or none) | (36) |  |
|  | **Co-coverage** = Any ANC + 3+ANC + first ANC in first trimester + BP measurement during pregnancy + Hb measurement during pregnancy + institutional delivery + PNC within 24 hours of delivery | - Score ranges from 0-7 | (43) |  |
|  | **Co-coverage of postnatal care** = umbilical cord check + temperature measurement + counseling on danger signs in the newborn + breastfeeding counseling + observation of breastfeeding | - Score ranges from 0-5 - In one of the papers, considers “appropriate newborn care” as receipt of 4-5 interventions | (52, 53) |  |
|  | **Co-coverage receiving food and cash during pregnancy** = received food during pregnancy + received cash during pregnancy or for birthing in health facility | - Count of 2 interventions - Binary outcome used (received 2 or none) | (72) |  |
|  | **Co-coverage receiving food and all interventions during pregnancy** = received food during pregnancy + received all 6 interventions during pregnancy (ANC4, weighted, 100+IFA, deworming, tetanus injections, health and nutrition counseling) | - Count of 2 linked intervention interventions (social protect and ANC interventions) - Count of receipt of 6 interventions - Binary outcome used (received 2 or none) | (72) |  |
|  | **Co-coverage receiving cash and food and all interventions during pregnancy** = received food and cash during pregnancy + received all 6 interventions during pregnancy (ANC4, weighted, 100+IFA, deworming, tetanus injections, health and nutrition counseling) | - Count of 2 linked intervention interventions (social protect and ANC interventions) - Count of receipt of 6 interventions, weighted - Binary outcome used (received 2 or none) | (72) |  |
|  | **Co-coverage receiving food and cash during childhood** = received food for children and received cash for completing vaccination for children | - Count of 2 interventions - Binary outcome used (received 2 or none) | (72) |  |
|  | **Co-coverage receiving food and all interventions during childhood** = received food and all interventions (IFA, VitA, deworming, counseling on child feeding, height and weight measurement) | - Count of 2 interventions - Binary outcome used (received 2 or none) | (72) |  |
|  | **Co-coverage receiving cash and all interventions during childhood** = received cash and all interventions (IFA, VitA, deworming, counseling on child feeding, height and weight measurement) | - Count of 2 linked intervention interventions (social protect and ANC interventions) - Count of receipt of 6 interventions - Binary outcome used (received 2 or none) | (72) |  |
|  | **Co-coverage receiving food and cash and all interventions during childhood** = received food and cash and all interventions (IFA, VitA, deworming, counseling on child feeding, height and weight measurement) | - Count of 2 linked intervention interventions (social protect and ANC interventions) - Count of receipt of 6 interventions - Binary outcome used (received 2 or none) | (72) |  |
|  | **Co-coverage** = receiving all standard maternal and child interventions except for family planning | - Score of all standard maternal and child interventions except for family planning - Considered cut points of <3 or 5+ interventions | (37) | Authors include a list of interventions measured; however, it is unclear which exactly were included in the final co-coverage measure. |
|  | **Access to adequate maternal healthcare** = women with at least 4 ANC visits with at least 1 being with a medically trained personnel + institutional delivery | - Coded as 1 if all requirements are met and 0 if otherwise | (69) |  |
|  | **Quality of ANC** = BP measurement + urine test + blood test + tetanus vaccines (at least 2 doses) + IFA | - Score from 0-5 - Adequate quality = 5 interventions received - Inadequate quality if <5 interventions received | (68) |  |
|  | **ANC Quality** = Among women who had at least 1 ANC visit, receipt of BP measurement + urine testing + blood testing | - Coded as 1 if all 3 interventions were received and as 0 if otherwise | (74) |  |
|  | **ANCQ** = blood test + urine test + BP measured (among women with a birth in the last 3 years that received at least one ANC visit) | - Binary outcome | (54) |  |
|  | **Quality Score for ANC** = Proportion of interventions received out of the following 16 interventions: ANC as per protocol, iron taken, iron taken for ≥180 days, told to look for possible pregnancy problems, told where to go if problems, told to get a postnatal checkup, advice for SBA delivery, advice for health facility delivery, ANC at health facilities, ANC by skilled providers, full neonatal tetanus protection, Albendazole is taken during pregnancy, BP measured in ANC visits, urine test in pregnancy, blood test in pregnancy, prepared at least 4 birth preparedness items out of 7 | - Continuous outcome as proportion of the 16 interventions received (proportion ranging from 0-1) | (57) |  |
|  | **Quality Score for institutional delivery** = Proportion of interventions received out of the following 8 interventions: delivery by skilled health personnel, received cash incentive, injection oxytocin labor and after delivery, counseling for injection oxytocin labor and after delivery, discharged from facility 12 hours after birth, dried before placenta out, wrapped before placenta out, skin-to-skin contact immediately | - Continuous outcome as proportion of the 8 interventions received (proportion ranging from 0-1) | (57) |  |
|  | **Quality Score for postnatal care** = Proportion of interventions received out of the following 11 interventions: PNC of mothers within 2 days, PNC of newborns within 2 days, PNC newborns by trained health workers, PNC of newborns at health facility, examined cord care, measured temperature, counselling newborn danger signs, counselled on breastfeeding, observed breastfeeding, observed for danger signs, bathing 24 hours after birth | - Continuous outcome as proportion of the 11 interventions received (proportion ranging from 0-1) | (57) |  |
|  | **Quality of care for newborn care** = count of receipt of the following 11 interventions: BCG vaccination + first Polio vaccination + not given prelacteal feed + early initiation of BF + temperature measured + counselling on danger signs + cord examined + counselling on BF + observation of BF + newborn weighted + skin-to-skin contact | - “High quality” if woman had a score >5 - Also consider quality-adjusted newborn PNC/Effective coverage, defined as % of newborns with PNC within 48 hours who met >6 of the contents of care (high quality care) | (67) |  |
|  | **Completeness of coverage and content of ANC services** = number of ANC visits + receipt of 7 ANC services: BP measured + iron supplementation + tetanus toxoid + explanations of pregnancy complications + urine test + blood test + weight measurement | - Categorical outcome: - Good (frequency of visits ≥4 and 7 complete components - Less (frequency of visits <4 and 7 complete components) - Less (frequency of visits ≥4 and 7 components were incomplete) - Poor (frequency of visits <4 and 7 components were incomplete - Also report a completeness of ANC components binary indicator as complete (7 components) and incomplete (7 components) | (55) |  |
|  | **Co-coverage of 8 clinical preventive services** = BP measurement, full protection against tetanus, first ANC visits at <4 months gestation, urine testing, counseling about danger signs, HIV counseling and testing, IFA supplementation for ≥ 90 days, at least 2 doses of SP | - Proportion of women with ≥ 4 ANC visits reporting receipt of the 8 clinical preventive services - Proportion of all women receiving all 8 interventions | (11) | Authors also calculate a simple average across this set of 8 interventions – see below in the simple average calculation category. |
|  | **Quality-adjusted coverage of ANC** = Among women with a live birth in the last 3 years who had 4+ ANC visits, those who received the following ANC components: BP taken, urine sample, blood sample, given or bought iron tablet/syrup | - Binary outcome coded as yes (1) for receiving all 4 services and as no (0) otherwise | (48) |  |
|  | **Quality-adjusted coverage of PNC** = Women with a live birth in the last 3 years who received PNC for the newborn within 24 hours, and the newborn was weighed and received BCG vaccination | - Binary outcome coded as yes (1) for being weighed and receiving BCG and no (0) otherwise | (48) |  |
|  | **Quality coverage of ANC** = % of Women with ≥1 ANC visit + all 8 ANC processes (Weight and height measured, BP measured, urine and blood tests, counselling for breastfeeding, danger signs, and birth preparedness) | - Continuous outcome - Percent of women with at least 1 ANC visit plus receipt of 8 ANC services | (13) |  |
|  | **Coverage of Quality Post-Partum Care** = % of women who had a post-partum check within 48 hours and met 5 post-partum processes (breasts and bleeding checked, counselled on danger signs, nutrition, and family planning) | - Continuous outcome - Percent of women with PNC within 48 hours plus receipt of 5 post-partum services | (13) |  |
|  | **Quality coverage of postnatal care** = % of newborns with PNC within 48 hours and meeting all 5 postnatal processes (Weighing newborn, checking cord care, counseling caregiver on breastfeeding, thermal care, and danger signs) | - Continuous outcome - Percent of newborns with timely PNC and receiving 5 services | (13) |  |
|  | **Effective ANC/Full Coverage of ANC Quality** = BP and weight measured, urine and blood samples taken at least once, tetanus vaccination, counseling about pregnancy danger signs | - Binary outcome | (59) |  |
|  | **Effective PNC/Full Coverage of PNC Quality** = polio and TB vaccination received by newborn and woman receiving a post-delivery health check by a provider | - Binary outcome | (59) |  |
|  | **Adequacy of ANC Examination** = If a woman was examined for 6 physical signs during first ANC visit (oedema, anemia, listen to foetal heart, weight, abdominal palpation, measure BP) | - Binary outcome of adequate examination (examined for 6 signs) or inadequate examination (examined for <6 signs) | (17) |  |
|  | **Adequacy of ANC Screening** = If a woman received 4 recommended tests during her first ANC visit (urine protein, syphilis, haemoglobin, voluntary counselling and testing for HIV) | - Binary outcome of adequate screening (received 4 tests) or inadequate screening (received <4 tests) | (17) |  |
|  | **Adequacy of ANC Interventions** = If a woman was offered 3 interventions in her 3^rd^ trimester (IEC on birth preparedness, fansidar/SP given for intermittent preventive treatment, and IFA) | - Binary outcome of adequate interventions (offered 3 interventions) or inadequate interventions (offered <3 interventions) | (17) |  |
|  | **ANC laboratory studies and exams summary measure** = 3 criteria: 1) complementary exams routinely recommended according to trimesters of gestation (first trimester = cervical colpocytology and lab exams: blood typing, serology for HIV, syphilis, and Hep B; stool culture test; CBC; urine culture and fasting blood glycemia; third trimester: serology for syphilis and AIDS and if necessary, toxoplasmosis and Hep B, urine culture and fasting blood glycemia; in first, second, or third trimester: at least one ultrasound); 2) clinical breast exam during gestational period, and 3) at least 1 ultrasound any time in high risk gestation or from follow-up until 17^th^ week | - Adequate ANC = all 3 criteria are met - Inadequate ANC = any of the 3 criteria is not met | (10) | 3 components are considered in the summary measure, but the first component (complementary exams routinely recommended according to trimester of gestation) includes consideration of multiple different laboratory tests and exams; therefore, more than 3 interventions are ultimately included. |
|  | **Independent Adequate content of ANC coverage =**Receiving at least 6 of the following interventions: weight measurement, blood pressure and abdominal examinations, urine, blood and HIV test, immunization against tetanus, and prescription of vitamins/mineral supplementation | - Binary outcome | (23) |  |
|  | **Conditional Adequate content of ANC coverage =** one of the following: 1) received ANC from either doctor, nurse, midwife, or others 2) received ANC from doctor or nurse 3) first ANC visit during the first trimester of pregnancy, 4) at least 4 ANC visits, and then received at least 6 of the following interventions: weight measurement, blood pressure and abdominal examinations, urine, blood and HIV test, immunization against tetanus, and prescription of vitamins/mineral supplementation. | - Binary outcome | (23) |  |
|  | **Standardized Comprehensive Antenatal and postnatal care =** (A) received ANC from either doctor, nurse, midwife or others, (B) received ANC from doctor or nurse, (C) first ANC visit during the first trimester of pregnancy, (D) at least four ANC visits, (E) Receiving at least 6 of the following interventions: weight measurement, blood pressure and abdominal examinations, urine, blood and HIV test, immunization against tetanus, and prescription of vitamins/mineral supplementation, (F) delivery attended by a doctor or nurse, (G) if  the newborn received a postnatal consultation within the first month of life. | - Binary outcome | (23) |  |
|  | **Complete Maternal Care (co-coverage)** = Women who received 4+ ANC visits and delivered in a facility | - Categorical outcome: If receiving 4+ ANC visits and delivering in a facility, considered to have complete maternal care; otherwise, women were assigned to one of the following groups: (i) no ANC & suboptimal delivery care, (ii) some ANC & suboptimal delivery care, (iii) complete ANC & suboptimal delivery care, (iv) no ANC & facility delivery care, or (v) some ANC & facility delivery care. | (26) |  |
|  | **Content of ANC care among women who received recommended ANC** = percentage of the following 8 components received: weighed, BP measured, urine or blood samples taken, discussed pregnancy complications, iron supplementations, drugs for intestinal parasites, malaria prophylaxis | - Women received a score as the % of 8 ANC components received | (26) |  |
|  | **Content of delivery care among women who delivered in health facilities** = percentage of the following 4 components received: initiating BF within 1 hour, weighing the baby, length of stay in facility post-delivery, and if the women was checked on prior to discharge | - Women received a score as the % of 4 components received | (26) |  |
|  | **Access to Adequate Care for Children** = Child was born in a public or private health facility + child received any vaccinations + child’s mother had access to prenatal care + child’s more received prenatal care from qualified staff + child’s mother reported 4+ANC visits | - Binary outcome of 1 if all conditions are met and 0 if otherwise | (22) |  |
|  | **ANC Quality/Quality-adjusted ANC** = proportion of women receiving the following 5 services: BP monitoring + iron supplementation + counselling about pregnancy complications + urine and blood testing among those with 4+ ANC visits from a skilled provider | - Binary outcome | (38, 59) |  |
|  | **Quality of Delivery Care/Quality-adjusted Delivery care** = institutional delivery/delivery at a health facility and receiving a post-partum check | - Binary outcome | (38, 58) |  |
|  | **Complete Continuum of Care** = women who received adequate ANC + institutional delivery + PNC + child was taken for full immunization | - Binary outcome | (52) |  |
|  | **Receiving PNC counseling services at least once** = counseling on early initiation and exclusive breastfeeding + use of IFA supplements + nutritional diet + postpartum family planning | - Binary outcome | (70) |  |
|  | **Received all 8 PNC services at least once** = body temperature + BP + vaginal examination for bleeding + uterus assessment + urinary problem assessment + breast examination + bowel function + perianal area examination | - Binary outcome | (70) | Calculated separately for i) within 24 hours after birth, ii) beyond 24 hours after birth, and iii) both within and beyond 24 hours after birth. |
|  | **Effective essential PNC services** = receiving all 4 counseling services ≥1 time during postnatal period (counseling on early initiation and exclusive breastfeeding + use of IFA supplements + nutritional diet + postpartum family planning) + receiving 8 essential PNC services both within 24 hours of birth and beyond 24 of birth (body temperature + BP + vaginal examination for bleeding + uterus assessment + urinary problem assessment + breast examination + bowel function + perianal area examination) | - Binary outcome | (70) |  |
|  | **Combined MDG access** = Having both improved water + improved sanitation | - Binary outcome of combined MDG access or not | (21) |  |
|  | **Combined SDG access** = Having both improved water with collection time < 30 min. + sanitation and a handwashing facility with soap | - Binary outcome of combined SDG access or not | (21) |  |
| ***Scoring System*** | **ANC Score** = Each recommended ANC practice is assigned a point value and based on receipt of the practice and repetition (if applicable); the points are added to generate a final score. The scoring is as follows: Antenatal visits = 5 points (6 repetitions for a total possible 30 points); Tetanus immunization = 1 point; Hep B immunization = 1 point; Influenza immunization = 1 point; TDAP immunization = 1 point; Ferrous sulfate prescription = 1 point; Folic acid prescription = 1 point; Dental exam = 1 point; educational activities = 1 point; CBC = 1 point; blood type = 1 point; fasting blood glucose = 1 point; stool parasitology = 1 point; urinalysis = 1 point (2 repetitions for a total possible 2 points); urine culture = 1 point (2 repetitions for a total possible 2 points); toxoplasmosis test = 1 point; syphilis test = 1 point (3 repetitions for a total possible 3 points); HIV test = 1 point (3 repetitions for a total possible 3 points); Hep B test = 1 point; OGTT 75g = 1 point; ultrasonography exam = 1 point | - Dichotomous outcome of “High” ANC score if ≥42 points (highest possible score is 56) | (19) |  |
|  | **ANCq** = The sum of the points from receipt of 7 different services, with each service assigned a point value as follows: first ANC visit in first trimester (1 point) + at least one visit with a skilled provider (2 points) + total number of ANC visits (1 point for 1-3 visits, 2 points for 4-7 visits, 3 points for 8+ visits) + BP measured (1 point) + blood sample taken (1 point) + urine sample taken (1 point) + receiving at least 2 tetanus toxoid injections (1 point) | - Score ranges from 0-10 | (49) |  |
| ***Linear mean (Simple Average)*** | **Composite Prevention Index** = average value of the following 9 indicators: 4+ ANC, exclusive breastfeeding, FP, improved water, adequate sanitation, BCG, DPT3, Polio immunizations | - Continuous outcome | (33) |  |
|  | **Composite Treatment Index** = average value of the following 4 indicators: institutional delivery, SBA, ORT, care-seeking for pneumonia | - Continuous outcome | (33) |  |
|  | **Overall MCH service coverage** = average of 4+ANC + facility + care-seeking for child diarrhea + care-seeking for pneumonia + care-seeking for fever | - Continuous outcome | (38) |  |
|  | **Water and sanitation coverage score** = ½(SW+ST)*100% | - Continuous outcome | (45) | Score was averaged with the maternal and newborn care coverage score and growth monitoring to create an overall coverage score. |
|  | **Overall coverage score** = 1/3(water and sanitation coverage score + maternal and newborn care coverage score + growth monitoring) | - Continuous outcome | (45) | While this score contains 3 components, the water and sanitation coverage score is a simple average of 2 components, and the maternal and newborn care coverage score is a weighted average of 3 components. |
|  | **Composite indicator of the primary outcomes of the trial** = (fully-immunized children + ORS use for diarrhea + sanitation index (a score based on drinking water index, food index, and domestic household hygiene index))/3 | - Continuous outcome | (65) | The following indices were included in the composite indicator and were calculated separately by adding the behaviors (with each item having a score of 1 or 0):  -Drinking water index = interior water container water is covered + exterior water container is clean + container contains water  -Food index = dishes are covered + clean dishes are stored high + all food is covered  -Domestic household hygiene index = no trash in yard + no trash in home + no unrestrained animals + no dirty clothes accumulated in the home + insignificant quantity of flies in the home + no standing water on the home patio/ |
|  | **Co-coverage** (at least 5 of 8 interventions) = BP measurement, full protection against tetanus, first ANC visits at <4 months gestation, urine testing, counseling about danger signs, HIV counseling and testing, IFA supplementation for ≥ 90 days, at least 2 doses of SP | -Continuous outcome  -Considered mean receipt of the interventions among women with 4+ ANC visits and among all women with a birth in the last 2 years | (11) | Calculate the proportion of women receiving all 8 interventions – see above in the count calculation category. |
| ***Weighted linear mean*** | **Composite Coverage Index (CCI)** = ¼(FPS+(SBA+ANCS)/2+(2DPT3+MSL+BCG)/4 + (ORT+CPNM)/2) | -Continuous outcome  -Dichotomized variable: partial coverage = CCI < 0.50; optimal coverage = CCI ≥ 0.50 | (5, 8, 14, 15, 1, 25, 28, 30, 33, 34, 35, 44, 46, 47, 50, 64, 71) | Some authors used at least one ANC visit, whereas others used 4+ ANC visits for the ANCS component of the formula. |
|  | **CCI** = ¼(FPCmo + (ANC4+SBA)/2 + (BCG+2DPT3+MSL)/4 + CAREANYD) | - Continuous outcome | (63) |  |
|  | **CCI** = 1/3(FPS + (SBA+ANCS)/2 + (2DPT3+MSL+BCG)/4) | - Continuous outcome | (39) |  |
|  | **Coverage Gap Index (CGI)** = 100%-1/4((ORT+ARI/2) + FP + (SBA+ANC/2) + (MSL+2DPT3+BCG/4)) | - Continuous outcome | (4, 6, 7, 42) |  |
|  | **Modified CCI** = 1/3((SBA+ANC1/2) + (2DTP3+BCG+MSL/4) + (ORT+CAREP/2)) | - Continuous outcome | (27, 37) | Excluded FPS due to data availability |
|  | **Modified CCI** = 1/4(FPS + (SBA+ANCS)/2 + (2DPT3+MSL)/3 + (ORT+CPNM)/2) | -Continuous outcome | (29) | Excluded BCG due to data availability |
|  | **Modified CCI** = 1/3((2DPT3+MSL+BCG)/4 + (VitA+TT+ANC)/3 + (SBA+AIW)/2) | - Continuous outcome | (73) |  |
|  | **Modified CCI** = 1/5((ANC+TTN)/2 + (FD+SBA)/2 + (BCG+Penta+Polio)/3 + FP | - Continuous outcome | (32) | Modified the CCI to assess MNCH service utilization, focusing on preventive MNCH interventions |
|  | **Modified CCI** = 1/6((ANC+TTN)/2 + (FD+SBA)/2 + PNCM + AABF + (BCG+2DPT3+2PL+MSL)/6 + FP) | - Continuous outcome | (41) | Modified CCI represents all 11 essential MNCH interventions and 6 stages of the continuum of care |
|  | **Modified CCI** = 1/6((ANC+TTN)/2 + (FD+SBA)/2 + (PNCM+PNCN)/2 + (BCG+2DPT3+2PL+MSL)/6 + AABF + FP) | - Continuous outcome | (66) | Modified CCI represents the continuum of care and includes the WHO’s recommended care components |
|  | **Modified ASHA-centric CCI (MACCI)** = 0.25*(0.33*[Complete ASHA home visits during antenatal period + full ANCS + FD] + [Complete HBNC] + 0.5*[DPT3+EBF] + 0.33*[Care seeking for neonatal complications + ORT + ARI/febrile illness]) | - Continuous outcome | (31) | Modified the CCI to measure the effectiveness of an intervention |
|  | **Maternal and newborn care coverage score** = 1/4(2*ANC4+HD+PNC)*100% | - Continuous outcome | (45) | This score was averaged with the water and sanitation coverage score and growth monitoring to create an overall coverage score. |
|  | **Child Immunization Coverage Score** = 1/8(BCG+MLS+2*DPT3+2*HB3+2*PLO3)*100% | - Continuous outcome | (45) |  |
|  | **Revised CCI** =1/4(DFPSmo + (ANC4+SBA)/2 + (MSL+BCG+2DPT3)/4 + (ORS+CAREP)/2) | - Continuous outcome | (47) | The revised CCI includes at least 4 ANC visits instead of at least 1 and demand for family planning satisfied with modern methods instead of just demand for family planning satisfied. |
|  | **Revised CCI + early BF initiation** = 1/5(DFPSmo + (ANC4+SBA)/2 + EIBF + (MSL+BCG+2DPT3)/4 + (ORS+CAREP)/2) | - Continuous outcome | (47) |  |
|  | **Revised CCI + early BF initiation + tetanus toxoid for pregnant women** = 1/5(DFPSmo + (ANC4+SBA)/2 + (EIBF+TET)/2 + (MSL+BCG+2DPT3)/4 + (ORS+CAREP)/2) | - Continuous outcome | (47) |  |
|  | **Revised CCI + early BF initiation + postnatal care for babies** = 1/5(DFPSmo + (ANC4+SBA)/2 + (EIBF+PNCALL)/2 + (MSL+BCG+2DPT3)/4 + (ORS+CAREP)/2) | - Continuous outcome | (47) |  |
|  | **Revised CCI + early BF initiation + tetanus toxoid for pregnant women + postnatal care for babies** = 1/5(DFPSmo + (ANC4+SBA)/2 + (EIBF+TET+PNCALL)/3 + (MSL+BCG+2DPT3)/4 + (ORS+CAREP)/2) | - Continuous outcome | (47) |  |
|  | **Maternal service CCI**= 1/3((ANC4+EANC+TT+HIVtest+FeFol)/5 + (SBA+HFdel)/2 + (PNC+EPNC)/2) | - Continuous outcome | (61) | Used overall mean as a cutoff point for a dichotomous outcome for logistic regression. |
| ***Geometric Mean*** | **UHC Index** = Service Coverage^0.5^ * Financial Protection^0.5^  (where service coverage is calculated by an unequally weighted geometric average of treatment (C-section rate, hospital delivery, SBA, use of outpatient care, hospital admissions) and prevention indicators (4+ANC, breast or cervical cancer screening, PNC, immunization, EBF, safe drinking water, adequate sanitation, physical access to health facility, non-use of tobacco) with a higher weight given to the prevention domain and financial protection is a simple average of 3 indicators of financial protection (health insurance coverage, no self-discharge for financial reasons, no catastrophic health expenses) | - Continuous outcome | (62) |  |
|  | **Composite Score** = [Full ANX(X1)*Institutional Delivery(X2)*Postnatal Visits(X3)*Full Immunization(X4)*Contraceptive Prevalence Rate(X5)*ORS Use Rate(X6)*Curative Care from Qualified Provider(X7)*Met Need Curative Care(X8)*Poverty Impact(@$1.25 (PPP))(X9)*Catastrophic Health Expenditure(X10)]^1/10^ | - Continuous outcome | (20) | Before inputting indicators into the index formula, they rescaled coverage indicators so that their values varied from 0-1. See the paper’s supplementary files for more information. |
| ***Principal Component Analysis*** | **Composite Score/Index=** (Full ANC *w_1_+ Institutional Delivery *w_2_+ Contraceptive Prevalence Rate *w_3_+ Full Immunization *w_4_+ Postnatal visits *w_5_) + (Poverty impact (@ $1.25 (PPP))*w_6_+ Catastrophic Health expenditure *w_7_) + (Curative care from Qualified provider *w_8_+ Met need curative care *w_9_) + (ORS use rate *w_10_).  *Where, w_1,_ w_2,…………………………….,_ w_10_ represents the final PCA weights used* | - Continuous outcome | (20) | See the paper’s supplementary files for additional details on how they used PCA. |
|  | **ANC Component Coverage iIndex=** PCA was used to estimate the index, which included the following dichotomous indicators: women’s weight, height, and BP, fundal weight, fetal heartbeat, blood test for HIV and syphilis, external and internal pelvic examination, receiving 90 IFA pills, 2 tetanus toxoid injections, information on pregnancy complication signs and what to do if there are signs, child sex, first child, mother’s education, mother’s age, monthly household expenditure, asset ownership | - Continuous outcome | (24) | Kaiser-Mayer-Olkin (KMKO) index and Bartlett’s sphericity test (p value <0.001) were used to assess if variables could be summarized via PCA. See the paper’s methods section for additional details regarding criteria applied. |
| ***Regression Weighting*** | **Composite Score** = b0+(*Full ANC**b1)+(*Institutional Delivery**b2)+(*Postnatal visits**b3)+(*Full Immunization**b4)+(*Contraceptive Prevalence Rate**b5)+(*ORS use rate**b6)+(*Curative care from Qualified provider**b7)+(*Met need curative care**b8)+(*Poverty impact (@$1.25 (PPP))**b9)+(*Catastrophic Health expenditure**b10)    Where, CS = composite score, b0 = Constant,  b1, b2, ...............................b10 = Regression coefficients (or weights) | - Continuous outcome | (20) | See paper’s supplementary files for additional details on how they used regression weighting. |
| ***Percentage Difference*** | **Percentage Difference (D%)** = Calculated the total percentage difference (D%) by summing the % coverage difference between 2 timepoints for all indicators of interest (14 indicators: contraceptive prevalence, 4+ANC, 2+ tetanus toxoid vaccination during last pregnancy, IPT2 malaria prophylaxis, SBA, 1+Postnatal visit, slept under LLIN/ITN night of survey, VitA, DPT3, full vaccination, U5 slept under LLIN/ITN night of survey, U5 diarrhoea treatment with ORS, U5 ARI treatment with appropriate antibiotics, and U5 fever treatment with appropriate anti-malarial) . Used the total percentage difference to compute Resilience Index =  $\frac{\sum_{1}^{14} {D\%}_{per system}-min\sum_{1}^{14} {D\%}_{across systems}}{\max\sum_{1}^{14} {D\%}_{across systems}-min\sum_{1}^{14} {D\%}_{across systems}}$ | - Components of resilience index formular | (40) | Tested 3 different definitions of resilience: 1) maintaining health system function, 2) improving health system function, and 3) achieving health system’s targets. See the paper for more information. |

**ANC**: Antenatal care, **ANC4**: 4 or more Antenatal Care visits, **ARI**: Acute respiratory infection, **BCG**: Bacille Calmette-Guerin vaccine, **CS**: Composite score, **CPNM:** care seeking for pneumonia, **CCI: Composite Coverage Index, CGI: Coverage Gap Index,** **DPT3**: Diphtheria, pertussis, and tetanus vaccine, **FP**: Family planning, **HB3**: Hepatitis B vaccine, **HIV**: Human immunodeficiency virus, **IFA**: Iron folic acid, **IPT2**: Intermittent preventive treatment, **ITN**: Insecticide-treated net, **LLIN**: Long-lasting insecticidal net, **MSL**: Measles vaccination, **MCH**: Maternal child health, **OGTT**: Oral glucose tolerance test, **ORS**: Oral rehydration solution, **PCA**: Principal component analysis, **PLO3**: Polio vaccination, **PNC**: Post natal care, **PPP**: Purchasing Power Parity, **RMNCH**: Reproductive, maternal, newborn, and child health, **SBA**: Skilled birth attendant, **TT**: Tetanus, **TDAP**: Tetanus, diphtheria, and pertussis vaccination, **u5**: Under 5 (years of age), **UHC**: Universal health coverage, **VitA**: Vitamin A,**WASH**: Water, sanitation, and hygiene.

**Supplementary Table 4: Key limitations of co-coverage indices as reported in studies included.**

| **Limitations identified** | **Frequency (n)** | **Studies** |
| --- | --- | --- |
| Inter-interviewer variability | 1 | (52) |
| Inability to include some interventions due to measurement error | 1 | (4) |
| Lack of studies assessing validity of MNCH interventions co-coverage indicators | 1 | (36) |
| Potential inaccuracy of ANC cards | 1 | (19) |
| Co-coverage index does not identify bottlenecks | 1 | (34) |
| Reduced sample size for co-coverage calculation by focusing on births in last 2 years | 2 | (1, 56) |
| Respondents' understanding of survey questions & care received | 3 | (52, 53, 59) |
| Lack of transparency of specific interventions included in co-coverage index | 2 | (39, (47) |
| Missing data | 5 | (13, 1, 33, 55, 59) |
| Potential social desirability | 7 | (6, 18), 26, 41, 57, 68, 69) |
| Inadequate information and measures on quality, timing, & content of interventions | 21 | (10, 13, 20, 21, 23, 26, 38, 40, 41, 48, 50, 51, 52, 55, 56, 58, 59, 60, 64, 67, 68) |
| Potential recall bias | 35 | (4, 5, 6, 13, 15, 16, 18, 23, 24, 26, 28, 29, 31, 32, 36, 38, 41, 43, 45, 48, 50, 51, 52, 53, 54, 55, 57, 58, 59, 60, 64, 66, 67, 68, 69) |

**Supplementary References**

1.	Wehrmeister FC, Restrepo-Mendez M-C, Franca GV, Victora CG, Barros AJ. Summary indices for monitoring universal coverage in maternal and child health care. Bull World Health Organ 2016;94:903–12.

2.	Barros AJD, Victora CG. Measuring Coverage in MNCH: Determining and Interpreting Inequalities in Coverage of Maternal, Newborn, and Child Health Interventions. PLOS Medicine Public Library of Science; 2013;10:e1001390.

3.	Victora CG, Fenn B, Bryce J, Kirkwood BR. Co-coverage of preventive interventions and implications for child-survival strategies: evidence from national surveys. The Lancet 2005;366:1460–6.

4.	Boerma JT, Bryce J, Kinfu Y, Axelson H, Victora CG. Mind the gap: equity and trends in coverage of maternal, newborn, and child health services in 54 Countdown countries. Lancet 2008;371:1259–67.

5.	Barros AJD, Ronsmans C, Axelson H, Loaiza E, Bertoldi AD, França GVA, Bryce J, Boerma JT, Victora CG. Equity in maternal, newborn, and child health interventions in Countdown to 2015: a retrospective review of survey data from 54 countries. Lancet 2012;379:1225–33.

6.	Rai RK, Kumar C, Singh PK. District level coverage gap in Maternal, Newborn and Child Health care services in India. J Epidemiol Glob Health 2012;2:221–4.

7.	Kumar C, Singh PK, Rai RK. Coverage gap in maternal and child health services in India: assessing trends and regional deprivation during 1992-2006. J Public Health (Oxf) 2013;35:598–606.

8.	Singh PK, Rai RK, Kumar C. Equity in maternal, newborn, and child health care coverage in India. Glob Health Action 2013;6:10.3402/gha.v6i0.22217.

9.	Victora CG, Barros AJD, Malpica-Llanos T, Walker N. How within-country inequalities and co-coverage may affect LiST estimates of lives saved by scaling up interventions. BMC Public Health 2013;13 Suppl 3:S24.

10.	da Fonseca CRB, Strufaldi MWL, de Carvalho LR, Puccini RF. Adequacy of antenatal care and its relationship with low birth weight in Botucatu, São Paulo, Brazil: a case-control study. BMC Pregnancy Childbirth 2014;14:255.

11.	Hodgins S, D’Agostino A. The quality–coverage gap in antenatal care: toward better measurement of effective coverage. Glob Health Sci Pract 2014;2:173–81.

12.	Miller NP, Degefie T, Hazel E, Legesse H, Tolera T, Amouzou A. Coverage and equitability of interventions to prevent child mortality in rural Jimma and West Hararghe Zones, Oromia Region, Ethiopia. Ethiop Med J 2014;52 Suppl 3:37–45.

13.	Marchant T, Tilley-Gyado RD, Tessema T, Singh K, Gautham M, Umar N, Berhanu D, Cousens S, Schellenberg JRA. Adding Content to Contacts: Measurement of High Quality Contacts for Maternal and Newborn Health in Ethiopia, North East Nigeria, and Uttar Pradesh, India. PLOS ONE Public Library of Science; 2015;10:e0126840.

14.	Restrepo-Méndez MC, Barros AJD, Requejo J, Durán P, Serpa LA de F, França GVA, Wehrmeister FC, Victora CG. Progress in reducing inequalities in reproductive, maternal, newborn,’ and child health in Latin America and the Caribbean: an unfinished agenda. Rev Panam Salud Publica 2015;38:9–16.

15.	Akseer N, Bhatti Z, Rizvi A, Salehi AS, Mashal T, Bhutta ZA. Coverage and inequalities in maternal and child health interventions in Afghanistan. BMC Public Health 2016;16:797.

16.	Carvajal-Aguirre L, Amouzou A, Mehra V, Ziqi M, Zaka N, Newby H. Gap between contact and content in maternal and newborn care: An analysis of data from 20 countries in sub-Saharan Africa. J Glob Health 2017;7:020501.

17.	Koroma MM, Kamara SS, Bangura EA, Kamara MA, Lokossou V, Keita N. The quality of free antenatal and delivery services in Northern Sierra Leone. Health Research Policy and Systems 2017;15:49.

18.	McDougal L, Atmavilas Y, Hay K, Silverman JG, Tarigopula UK, Raj A. Making the continuum of care work for mothers and infants: Does gender equity matter? Findings from a quasi-experimental study in Bihar, India. PLOS ONE Public Library of Science; 2017;12:e0171002.

19.	Oliveira-Ciabati L, Vieira CS, Franzon ACA, Alves D, Zaratini FS, Braga GC, Sanchez JAC, Bonifácio LP, Andrade MS, Fernandes M, et al. PRENACEL - a mHealth messaging system to complement antenatal care: a cluster randomized trial. Reprod Health 2017;14:146.

20.	Prinja S, Gupta R, Bahuguna P, Sharma A, Kumar Aggarwal A, Phogat A, Kumar R. A composite indicator to measure universal health care coverage in India: way forward for post-2015 health system performance monitoring framework. Health Policy Plan 2017;32:43–56.

21.	Roche R, Bain R, Cumming O. A long way to go – Estimates of combined water, sanitation and hygiene coverage for 25 sub-Saharan African countries. PLOS ONE Public Library of Science; 2017;12:e0171783.

22.	Sanoussi Y. Measurement and analysis of inequality of opportunity in access of maternal and child health care in Togo. BMC Health Services Research 2017;17:699.

23.	Serván-Mori E, Contreras-Loya D, Gomez-Dantés O, Nigenda G, Sosa-Rubí SG, Lozano R. Use of performance metrics for the measurement of universal coverage for maternal care in Mexico. Health Policy Plan 2017;32:625–33.

24.	Triyana M, Shankar AH. The effects of a household conditional cash transfer programme on coverage and quality of antenatal care: a secondary analysis of Indonesia’s pilot programme. BMJ Open 2017;7:e014348.

25.	Victora CG, Barros AJD, França GVA, da Silva ICM, Carvajal-Velez L, Amouzou A. The contribution of poor and rural populations to national trends in reproductive, maternal, newborn, and child health coverage: analyses of cross-sectional surveys from 64 countries. Lancet Glob Health 2017;5:e402–7.

26.	Benova L, Dennis ML, Lange IL, Campbell OMR, Waiswa P, Haemmerli M, Fernandez Y, Kerber K, Lawn JE, Santos AC, et al. Two decades of antenatal and delivery care in Uganda: a cross-sectional study using Demographic and Health Surveys. BMC Health Services Research 2018;18:758.

27.	Garchitorena A, Miller AC, Cordier LF, Rabeza VR, Randriamanambintsoa M, Razanadrakato H-TR, Hall L, Gikic D, Haruna J, McCarty M, et al. Early changes in intervention coverage and mortality rates following the implementation of an integrated health system intervention in Madagascar. BMJ Glob Health 2018;3:e000762.

28.	Keats EC, Akseer N, Bhatti Z, Macharia W, Ngugi A, Rizvi A, Bhutta ZA. Assessment of Inequalities in Coverage of Essential Reproductive, Maternal, Newborn, Child, and Adolescent Health Interventions in Kenya. JAMA Netw Open 2018;1:e185152.

29.	Thomson DR, Amoroso C, Atwood S, Bonds MH, Rwabukwisi FC, Drobac P, Finnegan KE, Farmer DB, Farmer PE, Habinshuti A, et al. Impact of a health system strengthening intervention on maternal and child health outputs and outcomes in rural Rwanda 2005-2010. BMJ Glob Health 2018;3:e000674.

30.	Akseer N, Rizvi A, Bhatti Z, Das JK, Everett K, Arur A, Chopra M, Bhutta ZA. Association of Exposure to Civil Conflict With Maternal Resilience and Maternal and Child Health and Health System Performance in Afghanistan. JAMA Netw Open 2019;2.

31.	Modi D, Dholakia N, Gopalan R, Venkatraman S, Dave K, Shah S, Desai G, Qazi SA, Sinha A, Pandey RM, et al. mHealth intervention “ImTeCHO” to improve delivery of maternal, neonatal, and child care services-A cluster-randomized trial in tribal areas of Gujarat, India. PLoS Med 2019;16:e1002939.

32.	Sakuma S, Yasuoka J, Phongluxa K, Jimba M. Determinants of continuum of care for maternal, newborn, and child health services in rural Khammouane, Lao PDR. PLoS One 2019;14:e0215635.

33.	Zhang C, Rahman MdS, Rahman MdM, Yawson AE, Shibuya K. Trends and projections of universal health coverage indicators in Ghana, 1995-2030: A national and subnational study. PLoS One 2019;14:e0209126.

34.	Barros AJD, Wehrmeister FC, Ferreira LZ, Vidaletti LP, Hosseinpoor AR, Victora CG. Are the poorest poor being left behind? Estimating global inequalities in reproductive, maternal, newborn and child health. BMJ Glob Health [Internet] BMJ Publishing Group Ltd; 2020 [cited 2025 Oct 30];5. Available from: https://gh.bmj.com/content/5/1/e002229

35.	Bouilly R, Gatica-Domínguez G, Mesenburg M, Cáceres Ureña FI, Leventhal DGP, Barros AJD, Victora CG, Wehrmeister FC. Maternal and child health inequalities among migrants: the case of Haiti and the Dominican Republic. Rev Panam Salud Publica 2020;44:e144.

36.	Carvajal L, Wilson E, Harris Requejo J, Newby H, de Carvalho Eriksson C, Liang M, Dennis M, Gohar F, Simen-Kapeu A, Idele P, et al. Basic maternal health care coverage among adolescents in 22 sub-Saharan African countries with high adolescent birth rate. J Glob Health 2020;10:021401.

37.	Garchitorena A, Miller AC, Cordier LF, Randriamanambintsoa M, Razanadrakato H-TR, Randriamihaja M, Razafinjato B, Finnegan KE, Haruna J, Rakotonirina L, et al. District-level health system strengthening for universal health coverage: evidence from a longitudinal cohort study in rural Madagascar, 2014-2018. BMJ Glob Health [Internet] BMJ Publishing Group Ltd; 2020 [cited 2025 Oct 29];5. Available from: https://gh.bmj.com/content/5/12/e003647

38.	Hategeka C, Arsenault C, Kruk ME. Temporal trends in coverage, quality and equity of maternal and child health services in Rwanda, 2000-2015. BMJ Glob Health 2020;5:e002768.

39.	Hermann B, Sathiya Susuman A, Aristide Romaric B, Mwinonè Hervé H. Mothers’ Preventive Health Care Practices and Children’s Survival in Burkina Faso: Findings from Repeated Cross-sectional Household Surveys. CPoS [Internet] 2020 [cited 2025 Oct 30];45. Available from: https://www.comparativepopulationstudies.de/index.php/CPoS/article/view/323

40.	Odhiambo J, Jeffery C, Lako R, Devkota B, Valadez JJ. Measuring health system resilience in a highly fragile nation during protracted conflict: South Sudan 2011–15. Health Policy Plan 2019;35:313–22.

41.	Oh J, Moon J, Choi JW, Kim K. Factors associated with the continuum of care for maternal, newborn and child health in The Gambia: a cross-sectional study using Demographic and Health Survey 2013. BMJ Open 2020;10:e036516.

42.	Panda BK, Kumar G, Awasthi A. District level inequality in reproductive, maternal, neonatal and child health coverage in India. BMC Public Health 2020;20:58.

43.	Ramesh BM, Dehury B, Isac S, Gothalwal V, Prakash R, Namasivayam V, Halli S, Blanchard J, Boerma T. The contribution of district prioritization on maternal and newborn health interventions coverage in rural India. J Glob Health 2020;10:010418.

44.	Thapa J, Budhathoki SS, Gurung R, Paudel P, Jha B, Ghimire A, Wrammert J, Kc A. Equity and Coverage in the Continuum of Reproductive, Maternal, Newborn and Child Health Services in Nepal-Projecting the Estimates on Death Averted Using the LiST Tool. Matern Child Health J 2020;24:22–30.

45.	Wang Y, Liao R, Feng XL. Equity in Essential Maternal, Newborn, and Child Health Interventions in Northeastern China, 2008 to 2018. Front Public Health 2020;8:212.

46.	Wehrmeister FC, Barros AJD, Hosseinpoor AR, Boerma T, Victora CG. Measuring universal health coverage in reproductive, maternal, newborn and child health: An update of the composite coverage index. PLoS One 2020;15:e0232350.

47.	Wehrmeister FC, Fayé CM, da Silva ICM, Amouzou A, Ferreira LZ, Jiwani SS, Melesse DY, Mutua M, Maïga A, Ca T, et al. Wealth-related inequalities in the coverage of reproductive, maternal, newborn and child health interventions in 36 countries in the African Region. Bull World Health Organ 2020;98:394–405.

48.	Anindya K, Marthias T, Vellakkal S, Carvalho N, Atun R, Morgan A, Zhao Y, Hulse ES, McPake B, Lee JT. Socioeconomic inequalities in effective service coverage for reproductive, maternal, newborn, and child health: a comparative analysis of 39 low-income and middle-income countries. EClinicalMedicine 2021;40:101103.

49.	Arroyave L, Saad GE, Victora CG, Barros AJD. Inequalities in antenatal care coverage and quality: an analysis from 63 low and middle-income countries using the ANCq content-qualified coverage indicator. International Journal for Equity in Health 2021;20:102.

50.	Gandhi S, Maharatha TM, Dash U, Babu M S. Level of inequality and the role of governance indicators in the coverage of reproductive maternal and child healthcare services: Findings from India. PLoS One 2021;16:e0258244.

51.	Kothavale A, Meher T. Level of completion along continuum of care for maternal, newborn and child health services and factors associated with it among women in India: a population-based cross-sectional study. BMC Pregnancy and Childbirth 2021;21:731.

52.	Peven K, Day LT, Bick D, Purssell E, Taylor C, Akuze J, Mallick L. Household Survey Measurement of Newborn Postnatal Care: Coverage, Quality Gaps, and Internal Inconsistencies in Responses. Glob Health Sci Pract 2021;9:737–51.

53.	Peven K, Mallick L, Taylor C, Bick D, Day LT, Kadzem L, Purssell E. Equity in newborn care, evidence from national surveys in low- and middle-income countries. International Journal for Equity in Health 2021;20:132.

54.	Sanhueza A, Carvajal-Vélez L, Mújica OJ, Vidaletti LP, Victora CG, Barros AJ. SDG3-related inequalities in women’s, children’s and adolescents’ health: an SDG monitoring baseline for Latin America and the Caribbean using national cross-sectional surveys. BMJ Open 2021;11:e047779.

55.	Arsyi, Miftahul, Besral, Bersal, Herdayati, Milla, Phalkey, Revathy. Antenatal Care Services and Incidence of Low Birth Weight: A Comparison of Demographic and Health Surveys in 4 ASEAN Countries. Journal of Preventive Medicine & Public Health 2022;55.

56.	Baye K, Laillou A, Chitekwe S. Co-coverage of reproductive, maternal, newborn and child health interventions shows wide inequalities and is associated with child nutritional outcomes in Ethiopia (2005-2019). Matern Child Nutr 2022;20 Suppl 5:e13452.

57.	Khatri RB, Durham J, Karkee R, Assefa Y. High coverage but low quality of maternal and newborn health services in the coverage cascade: who is benefitted and left behind in accessing better quality health services in Nepal? Reproductive Health 2022;19:163.

58.	Kim MK, Kim SA, Oh J, Kim CE, Arsenault C. Measuring effective coverage of maternal and child health services in Cambodia: a retrospective analysis of Demographic and Health Surveys from 2005 to 2014. BMJ Open 2022;12:e062028.

59.	Lindberg C, Nareeba T, Kajungu D, Hirose A. The Extent of Universal Health Coverage for Maternal Health Services in Eastern Uganda: A Cross Sectional Study. Matern Child Health J 2022;26:632–41.

60.	Nguyen PH, Singh N, Scott S, Neupane S, Jangid M, Walia M, Murira Z, Bhutta ZA, Torlesse H, Piwoz E, et al. Unequal coverage of nutrition and health interventions for women and children in seven countries. Bull World Health Organ 2022;100:20–9.

61.	Worku AG, Tilahun HA, Belay H, Mohammedsanni A, Wendrad N, Abate B, Mohammed M, Ahmed M, Wondarad Y, Abebaw M, et al. Maternal Service Coverage and Its Relationship To Health Information System Performance: A Linked Facility and Population-Based Survey in Ethiopia. Glob Health Sci Pract 2022;10:e2100688.

62.	Zhou Y, Li C, Wang M, Xu S, Wang L, Hu J, Ding L, Wang W. Universal health coverage in China: a serial national cross-sectional study of surveys from 2003 to 2018. The Lancet Public Health Elsevier; 2022;7:e1051–63.

63.	Blanchard AK, Jacobs C, Musukuma M, Chooye O, Sikapande B, Michelo C, Boerma T, Wehrmeister FC. Going deeper with health equity measurement: how much more can surveys reveal about inequalities in health intervention coverage and mortality in Zambia? Int J Equity Health 2023;22:109.

64.	Cisse D, Toure AA, Diallo A, Goungounga JA, Kadio KJ-JO, Barry I, Berete S, Magassouba AS, Harouna SH, Camara AY, et al. Evaluation of maternal and child care continuum in Guinea: a secondary analysis of two demographic and health surveys using the composite coverage index (CCI). BMC Pregnancy and Childbirth 2023;23:391.

65.	Das JK, Salam RA, Rizvi A, Soofi SB, Bhutta ZA. Community Mobilization and Community Incentivization (CoMIC) Strategy for Child Health in a Rural Setting of Pakistan: Study Protocol for a Randomized Controlled Trial. Methods and Protocols Multidisciplinary Digital Publishing Institute; 2023;6:30.

66.	Gebremedhin AF, Dawson A, Hayen A. Determinants of continuum of care for maternal, newborn, and child health services in Ethiopia: Analysis of the modified composite coverage index using a quantile regression approach. PLoS One 2023;18:e0280629.

67.	Gebremedhin AF, Dawson A, Hayen A. Effective coverage of newborn postnatal care in Ethiopia: Measuring inequality and spatial distribution of quality-adjusted coverage. PLOS ONE Public Library of Science; 2023;18:e0293520.

68.	Girotra S, Malik M, Roy S, Basu S. Utilization and determinants of adequate quality antenatal care services in India: evidence from the National Family Health Survey (NFHS-5) (2019-21). BMC Pregnancy Childbirth 2023;23:800.

69.	Methun MIH, Ahinkorah BO, Roy S, Okyere J, Hossain MI, Haq I, Ume SS, Kamrul Hasan M. Inequalities in adequate maternal healthcare opportunities: evidence from Bangladesh Demographic and Health Survey 2017-2018. BMJ Open 2023;13:e070111.

70.	Tauhidi L, Mureed S, Raza TE, Hamid S, Hanif K, Emmanuel F. Measuring Coverage of Essential Maternal Postnatal Care Services in the Squatter Settlements of Islamabad Capital Territory in Pakistan. Int J Soc Determinants Health Health Serv 2023;53:294–302.

71.	Vidaletti LP, Cata-Preta BO, Phillips DE, Shekhar S, Barros AJD, Victora CG. Time trends in ethnic inequalities in child health and nutrition: analysis of 59 low and middle-income countries. International Journal for Equity in Health 2023;22:76.

72.	Nguyen PH, Avula R, Neupane S, Akseer N, Heidkamp R. Identifying measures for coverage of nutrition-sensitive social protection programs: Learnings from India. Matern Child Nutr 2024;20:e13661.

73.	S R, Neethi Mohan V, Vaidyanathan G, Dash U, Muraleedharan VR. Wealth and education-related inequalities in the utilisation of reproductive, maternal, newborn, and child health interventions within scheduled tribes in India: an analysis of Odisha and Jharkhand. BMC Public Health 2024;24:1605.

74.	Shibre G, Mekonnen W, Haile Mariam D. Explaining changes in educational disparities in competent maternal health care services in urban and rural areas in Ethiopia. Front Public Health [Internet] Frontiers; 2024 [cited 2025 Oct 30];12. Available from: https://www.frontiersin.org/journals/public-health/articles/10.3389/fpubh.2024.1332801/full
